# Use of genotyping-by-sequencing to determine the genetic structure in the medicinal plant chamomile, and to identify flowering time and alpha-bisabolol associated SNP-loci by genome-wide association mapping

**DOI:** 10.1186/s12864-017-3991-0

**Published:** 2017-08-10

**Authors:** Lars-Gernot Otto, Prodyut Mondal, Jonathan Brassac, Susanne Preiss, Jörg Degenhardt, Sang He, Jochen Christoph Reif, Timothy Francis Sharbel

**Affiliations:** 1 Apomixis Research Group, Department Plant Breeding Research, Leibniz Institute of Plant Genetics and Crop Plant Research (IPK), Corrensstrasse 3, D-06466 Seeland OT Gatersleben, Germany; 2 Research Group of Pharmaceutical Biotechnology, Institute of Pharmacy, Martin-Luther University Halle-Wittenberg, Hoher Weg 8, 06120 Halle (Saale), Germany; 3 Quantitative Genetics Research Group, Department Plant Breeding Research, Leibniz Institute of Plant Genetics and Crop Plant Research (IPK), Corrensstrasse 3, D-06466 Seeland OT Gatersleben, Germany; 4 Global Institute for Food Security, University of Saskatchewan, 110 Gymnasium Place, Saskatoon, SK S7N 4J8 Canada

**Keywords:** *Matricaria recutita*, Chamomile, Medicinal and aromatic plant (MAP), Genetic diversity, Genome-wide association study (GWAS), Bisabolol, Genetic resources, Genotyping by sequencing (GBS), Single nucleotide polymorphism (SNP), Structure

## Abstract

**Background:**

Chamomile (*Matricaria recutita* L.) has a long history of use in herbal medicine with various applications, and the flower heads contain numerous secondary metabolites which are medicinally active.

In the major crop plants, next generation sequencing (NGS) approaches are intensely applied to exploit genetic resources, to develop genomic resources and to enhance breeding. Here, genotyping-by-sequencing (GBS) has been used in the non-model medicinal plant chamomile to evaluate the genetic structure of the cultivated varieties/populations, and to perform genome wide association study (GWAS) focusing on genes with large effect on flowering time and the medicinally important alpha-bisabolol content.

**Results:**

GBS analysis allowed the identification of 6495 high-quality SNP-markers in our panel of 91 *M. recutita* plants from 33 origins (2–4 genotypes each) and 4 *M. discoidea* plants as outgroup, grown in the greenhouse in Gatersleben, Germany. *M. recutita* proved to be clearly distinct from the outgroup, as was demonstrated by different cluster and principal coordinate analyses using the SNP-markers. Chamomile genotypes from the same origin were mostly genetically similar. Model-based cluster analysis revealed one large group of tetraploid genotypes with low genetic differentiation including 39 plants from 14 origins. Tetraploids tended to display lower genetic diversity than diploids, probably reflecting their origin by artificial polyploidisation from only a limited set of genetic backgrounds.

Analyses of flowering time demonstrated that diploids generally flowered earlier than tetraploids, and the analysis of alpha-bisabolol identified several tetraploid genotypes with a high content. GWAS identified highly significant (*P* < 0.01) SNPs for flowering time (9) and alpha-bisabolol (71). One sequence harbouring SNPs associated with flowering time was described to play a role in self-pollination in *Arabidopsis thaliana*, whereas four sequences harbouring SNPs associated with alpha-bisabolol were identified to be involved in plant biotic and abiotic stress response in various plants species.

**Conclusions:**

The first genomic resource for future applications to enhance breeding in chamomile was created, andanalyses of diversity will facilitate the exploitation of these genetic resources. The GWAS data pave the way for future research towards the genetics underlying important traits in chamomile, the identification of marker-trait associations, and development of reliable markers for practical breeding.

**Electronic supplementary material:**

The online version of this article (doi:10.1186/s12864-017-3991-0) contains supplementary material, which is available to authorized users.

## Background

German Chamomile (*Matricaria recutita* L. syn. *Chamomilla recutita* (L.) Rauschert; from hereon chamomile) is described to be native to the Near East and South-Southeast Europe [1] and is grown in many temperate regions of the world. As already mentioned by Hippocrates (fifth century BC, [2]), it has a long history of use in herbal medicine, with applications in gastrointestinal diseases, treatment of infections and inflammatory diseases of the skin, and respiratory problems. The flower heads contain medicinal compounds, and are either used dried (e.g. tea) or the essential oils are extracted. Chamomile has numerous secondary metabolites which are medicinally active, including the terpenes alpha-bisabolol (syn. Levomenol) and its derivates (bisaboloids), matricine/chamazulene, further terpenes, and several flavonoids [2].

Chamomile is mainly outcrossing [3, 4], and as most chamomile varieties are rather landraces, they display a high degree of phenotypic variability reflecting that of natural populations [5], from which they were isolated. Whereas wild populations of chamomile are diploid, agronomically cultivated varieties are either diploid or artificially generated tetraploid, the latter of which have larger flower heads and higher 1000-seed weight [6]. Genetic diversity in chamomile was evaluated in a few studies using a variety of different genetic markers (summarized in [7]), including AFLP [8], RAPD [8, 9] and ISSR [10]. Next-generation sequencing (NGS) approaches have been successfully used for the analysis and exploitation of genetic and genomic resources in crop plant breeding, including rapeseed, soybean, barley, or maize, e.g. [11, 12]. One NGS approach, genotyping by sequencing (GBS), has been widely used in many crop plants (e.g. [13–18]), is highly reproducible [13] and reduces genome complexity with restriction enzymes, thus, lowering costs. In contrast, the use of NGS / GBS for the amelioration of medicinal and aromatic plant breeding is relatively underexploited. An NGS approach has not yet been described for the non-model plant species chamomile.

In our work, we applied GBS to a set of chamomile origins (varieties, populations, accessions) to identify genome-wide, high quality single nucleotide polymorphisms (SNPs), to estimate genetic diversity and to reveal genetic structure within our cultivated chamomile germplasm collection. Therefore this is the first study reporting the generation and use of SNP markers in chamomile. We furthermore phenotyped the plants for flowering time (FT) and the content of bisaboloids by gas chromatography–mass spectrometry (GC-MS). Using phenotyping and genotyping data, an association study was performed to identify SNPs associated with FT and bisaboloids content, and to search for possible candidate genes underlying FT and the production of the medicinally-important compound alpha-bisabolol. In comparison to other marker platforms like AFLP or RAPD, the high marker density of genome-wide SNPs generated by GBS increases the probability of identifying markers closely linked to genes of interest. The resulting data provide a basis to enhance breeding in chamomile.

## Methods

### Plant material

Ninety one genotypes, representing 2–4 genotypes each from 33 origins of *M. recutita* L., and an additional 4 genotypes from 2 *M. discoidea* DC accessions as outgroup, were included in the analysis (Table 1). The focus of the study on cultivated chamomile, mainly encompassing origins from all over Europe, but also including some locations outside of Europe.

**Table 1:** Origin name, genotype code, described geographical origin and source of the investigated plants

Origin^a^	Genotype code	Geographic origin	Geographic region^b^	Source
Manzana1	002_02^c^, 002_04^c^	Germany	1	Pharmaplant GmbH
Bodegold	003_01^c^, 003_02^c^, 003_03^c^, 003_04^c^	Germany	1	Pharmasaat GmbH
Camoflora	004_01^c^, 004_03^c^, 004_04^c^	Germany	1	Pharmaplant GmbH
Lutea	005_01^c^, 005_02^c^, 005_03^c^, 005_04^c^	Slovakia	2	Pharmaplant GmbH
Zloty Lan	006_01^c^, 006_02, 006_03^c^, 006_04^c^	Poland	2	Pharmasaat GmbH
Goral	007_01^c^, 007_02^c^, 007_03^c^, 007_04	Slovakia	2	Pharmasaat GmbH
Bohemia	008_02^c^, 008_04^c^, 008_05^c^	Czech Republic	2	Semo
Promyk	009_01, 009_02^c^, 009_03^c^	Poland	2	Pharmaplant GmbH
Argenmilla	010_02^c^, 010_03, 010_06^c^	Argentina	5	Pharmaplant GmbH
trade B + T	011_01^c^, 011_04^c^, 011_05^c^	France	1	B and T World Seeds
trade FStM	013_04, 013_06^cc^	France	1	Ferme de Saint Marthe
trade PNOS	014_04^c^, 014_05^c^	Poland	2	PNOS Poland
trade Kiepenkerl	016_02^c^, 016_04^c^	Germany	1	Kiepenkerl, Bruno Nebelung GmbH
trade Italy 6	019_01^c^, 019_03^c^	Italy	4	Flortis
pop England 1	020_01, 020_04	England	1	Pharmaplant GmbH
trade Garafarm	021_02^c^, 021_03^c^, 021_05^c^	Hungary	2	Garafarm
Margaritar	022_01^c^, 022_02^c^, 022_04^c^, 022_06^c^	Romania	3	Pharmaplant GmbH
trade Agbina	023_01^c^, 023_02^c^, 023_03^c^	Russia	5	Agbina
Lazur	024_02^c^, 024_03^c^, 024_05^c^, 024_07^c^	Bulgaria	3	Pharmaplant GmbH
Germania	026_01^c^, 026_02, 026_04^c^, 026_05^c^	Egypt	4	N.L. Chrestensen
trade USA	027_02^c^, 027_03, 027_04^c^	USA	5	Pharmaplant GmbH
pop Croatia	029_03^c^, 029_04^c^, 029_07^c^	Kroatien	3	Pharmaplant GmbH
trade Italy 5	032_01^c^, 032_04^c^, 032_05^c^	Italy	4	Flortis
trade Italy 4	033_03^c^, 033_05^c^	Italy	4	Plantania
pop Germany 1	064_01^c^, 064_03^c^	Germany	1	Genebank Gatersleben MAT 2
Pohorelicky Velkokvety	066_04^c^, 066_07^c^	Czech Republic	2	Genebank Gatersleben MAT 15
pop Bulgaria 1	067_02, 067_04	Bulgaria	3	Genebank Gatersleben MAT 16
Krajovy	515_01^c^, 515_04	Slovakia	2	Genebank Gatersleben MAT 10
pop North Korea 1	516_01, 516_04, 516_07^c^	North Korea	5	Genebank Gatersleben MAT 19
Bona	715_04^c^, 715_06	Slovakia	2	Vet.med. University of Vienna
Manzana2	717_03^c^, 717_04^c^	Austria	1	Vet.med. University of Vienna
pop England 2	721_03^c^, 721_04^c^	England	1	coll. R. Pickering
pop England 3	722_03^c^, 722_04^c^	England	1	coll. R. Pickering
*M. discoidea* 1	701_01^c^	Germany	1	Loki-Schmidt Genbank Osnabrück, Inv.-Nr:09–00-0145
M. discoidea 2	MD1_04^c^, MD1_05^c^, MD1_06^c^	Germany	1	Loki-Schmidt Genbank Osnabrück, Inv.-Nr:04–00-0280

^a^Origins (variety, accession, wild and cultivated population) are named, if applicable, with the same or consecutive numbers as in Otto et al. [19] (e.g. trade Italy 6)

^b^Based upon the climate from each origin, plants were classified into 5 geographic groups (according to Kottek et al. [76]): (1) middle-west Europe (2) middle-east Europe: (3) south-east Europe: (4) Mediterranean (5) remainder: USA, Argentina, Russia, North-Korea

^c^Analysis of chemical compounds was performed

For the purpose of this study, we define an origin as either a registered variety, a gene bank accession, a cultivated or wild population of chamomile. The term population applies to a wild natural population as well as to a cultivated one selected for breeding or agriculture. Genotype refers to a genetically individual plant.

### Plant cultivation and ploidy analysis

Seeds were sown in the greenhouse on the 16th July 2014, and seedlings were individually transplanted into single pots (14 cm diameter) after reaching the 20-leaf stage. Between 2 to 4 genotypes per origin were selected for the subsequent analysis, with a focus on elite varieties/populations (4 genotypes each). Plant ploidy was determined by flow-cytometry according to Otto et al. [19] at the 10- to 20-leaf stage.

Plants were randomized in the greenhouse, organized in a block of plants of 6 × 16 plants, and grown until the 31st of January 2015 under natural light conditions (short day conditions, with day / night temperatures of 17–21 °C (12 h) / 14–19 °C (12 h)) and from February 2015 until the end of April 2015 under additional light for 16 h (long day conditions, MASTER Agro 400 W E40 1SL Na-bulb lamp, 2000 K colour temperature, Royal Philips N.V. Netherlands) with day / night temperatures of 18–21 °C (12 h) / 15–17 °C (12 h). Flowering time (FT) was measured as days after sowing (DAS).

### Analysis of chemical compounds by gas chromatography - mass spectrometry (GC-MS)

The content (peak areas) of bisaboloids (terpenoids), namely bisabolol oxide B, bisabolon oxide A, alpha-bisabolol, and bisabolol oxide A, were analysed by gas chromatography for 80 samples (76 from *M. recutita* and 4 from *M. discoidea*). The content of the chemical compounds was determined for each genotype in a sample of pooled flower heads, where flower heads were harvested after the first 2 circles of florets started flowering, scored as the flowering time until maximum half of the florets were flowering. All plants were grown and processed equally. Samples were immediately frozen in liquid nitrogen and stored at −80 °C until further analysis to prevent the oxidation of alpha-bisabolol.

The analysis by GC-MS was done with modifications according to Köllner et al. [20] and Irmisch et al. [21]. For each sample, the finely ground powder on liquid nitrogen of 80 to 100 mg material from 5 flower heads was used. For each pulverized sample 200–400 μl of n-hexane was added, followed by overnight incubation with shaking at 25 °C. A total of 100 μl was transferred to a clean microtube fitted for GC vials. Terpene analysis was carried out with a gas chromatograph (GC 2010, Shimadzu, Duisburg, Germany) equipped with a splitless injector (injector temperature, 220 °C; injection volume, 1 μl) and coupled to a quadrupole mass selective detector (Shimadzu). H_2_ was used as carrier gas at a flow rate of 1 ml min-^1^. Samples were analysed on a Supreme-5 ms column (30 m length × 0.25 mm inner diameter × 0.25 μm film thickness, Chromatographie Service GmbH, Germany). The coupled mass spectrometer was operated with a transfer line temperature of 230 °C, a source temperature of 230 °C, a quadrupole temperature of 150 °C, an ionization potential of 70 eV and a scan range of 50–350 amu.

A flame ionization detector (FID) was used for the quantification of the compounds (μg/g of fresh weight), and operated at 250 °C. Compounds were identified by comparison of retention times and mass spectra to those of authentic reference substances obtained from Sigma-Aldrich (Steinheim, Germany), Roth (Karlsruhe, Germany), or by reference spectra in the Wiley and Shimadzu libraries [22].

### DNA extraction and genotyping-by-sequencing (GBS)

DNA was isolated from the 95 samples according to the manufacturer’s instruction using the Agencourt Chloropure kit (Agencourt Bioscience Corp., Beverly, Massachusetts, USA). After isolation the DNA was analysed on a 1.3% Agarose gel (Bio&SELL Universal-Agarose), and the DNA concentration was determined relative to a DNA standard. DNA dissolved in TE buffer was shipped to the Cornell University Biotechnology Resource Center (BRC, Cornell, USA) for GBS analysis.

Sample preparation and sequencing was performed at the BRC using a protocol modified from Elshire et al. [3] (http://www.biotech.cornell.edu/brc/genomics-facility) using the enzyme ApeKI for digestion. GBS libraries constructed were sequenced in the BRC Genomics Facility on the Illumina HiSeq 2000/2500 (100 bp, single-end reads).

### Sequencing data analysis and SNP detection

In the absence of a reference genome, the assembly was done de novo. The 156 × 10^6^ barcoded reads from the 95 samples (genotypes) were demultiplexed, trimmed for restriction site and barcode, filtered and clustered using the software pipeline pyRAD v. 3.0 [23]. If not described differently, the default settings were used. The 4 samples from *M. discoidea* were assigned as outgroup, with the 91 remaining samples also defined as threshold (*n* = 91) for the maximum number of individuals with a shared heterozygous site to account for the high proportion of polyploid samples and the outcrossing nature of *M. recutita*. The minimal number of samples per locus was set to 75, i.e. 75 from 91 samples having data at a particular locus, the parameter for the clustering threshold of reads within and between individuals was set to 0.88, and filtering for barcodes, adapters and cut sites was done strictly setting value “2”. The maximum ploidy level was appointed as tetraploid.

Using the above criteria, the pyRAD pipeline filtered and called 6495 SNPs from the 91 *M. recutita* genotypes.

### Analysis of the genetic diversity and genetic structure

The genetic diversity and structure of cultivated chamomile was analysed with the filtered 6495 high-quality SNPs using the admixture model of STRUCTURE 2.3.4 [24–26]. The outgroup (4 genotypes from *M. discoidea*) was excluded from the analysis, since *M. discoidea* was clearly different from *M. recutita*. Ten independent analyses with K (number of clusters) ranging from 1 to 15 with 15,000 iterations as burn-in were performed followed by 50,000 supplementary Markov Chain Monte Carlo (MCMC) iterations. The optimal K was obtained according to Evanno et al. [27] using STRUCTURE Harvester [28]. For K = 3 and K = 7, an additional 10 independent analyses with 25,000 iterations as burn-in followed by 100,000 supplementary MCMC iterations were done and used for further analysis. The burn-in length was assessed by monitoring the parameters. CLUMPP v 1.1.2 [29] was used to align the results of individual runs (Greedy algorithm).

The allele frequencies were assumed to be correlated between the origins of chamomile rather than independent [25], as chamomile is a mainly outcrossing species whose seeds are exchanged for cultivation and breeding between regions, and thus the investigated populations / origins can share similar allele frequencies [25]. The admixture model was used, since it is more general and does not assume each individual to belong to a single cluster [26]. The value for lambda (allele frequencies parameter) was set to 0.3 after lambda was estimated in STRUCTURE. Otherwise, the default settings of STRUCTURE were used.

Additionally, the supermatrix of concatenated sequences, 682,699 bp long, was used to compute a phylogenetic tree. The neighbor joining method [30] with 100 bootstrap and keeping nodes above 75% was used to obtain a consensus phylogenetic tree with Geneious v.10.0.9 (https://www.geneious.com, [31]). To validate the results from the neighbor-joining and STRUCTURE analysis, principal coordinate analysis (PCoA) was done.

### Analysis of phenotype-genotype associations

To obtain markers for GWAS analysis (genome wide association studies), the full sequences (contigs) identified by the pyRAD pipeline as harbouring all the 6495 SNPs of the STRUCTURE analysis were used to detect 44,468 polymorphic markers within a set incorporating all 95 genotypes. Due to the lack of genetic or physical map, the minor number of missing profiles were imputed by means of Random Forest algorithm [32] through R package “missForest” [33]. Quality control was applied such that markers with call rates less than 90% were excluded [34, 35]. In the end, out of the 44,468 polymorphic markers 16,972 markers, including 16,333 biallelic and 639 multiallelic, were available for subsequent GWAS. Since the single results in GWAS were subsequently evaluated (including consideration of the *p*-values and r^2^-values), different criteria for SNP filtering were applied than for the genetic structure analysis so that more SNPs could be used as basic data for the GWAS.

Considering the lack of phenotyping replicates here, estimating the heritability was not feasible. Nevertheless, genomic heritability [36, 37] is a well-suited surrogate for the heritability. We estimated genomic heritability via a replicated (20×) 5-fold cross validation, as the square of average correlation from the 20 replications comparing predicted genotypic values and phenotypic values of respective traits.

GWAS was performed with the R package “rrBLUP” [38] using phenotypic data and the 16,972 markers obtained. The kinship matrix was estimated with a G matrix [39] using 5768 markers (out of the total 16,972 markers) whose minor allele frequency was above 0.05. The final significance test for each marker/allele was accomplished using F-test in “rrBLUP”. The percentage of phenotypic variance explaining each significant marker was estimated via R^2^ by fitting a regression between phenotypes and marker profiles, as implemented in R using the function “lm”.

The DNA sequences containing highly significant markers as identified by GWAS were annotated using firstly BLAST (blastn and tblastx; [40, 41], http://blast.ncbi.nlm.nih.gov/Blast.cgi) against the NCBI plant genomes nucleotide collection of flowering plants data using the discontiguous megablast algorithm. Mapping and annotation of the BLAST-results were performed using Blast2GO 4.0.2 and default settings [42–45]. Additionally, blastn against the *Helianthus annuus* (sunflower) XRQ genome assembly was done at https://www.heliagene.org/HanXRQ-SUNRISE/ [46], since sunflower is the closest related species with a sequenced genome available, belonging together with chamomile in the Asteraceae. Local BLAST analysis was done with CLC Genomics Workbench 8.5 (QIAGEN Aarhus A/S).

## Results

### Analysis of genetic structure reveals less genetic diversity for tetraploid than for diploid chamomile origins

**Fig. 1:**
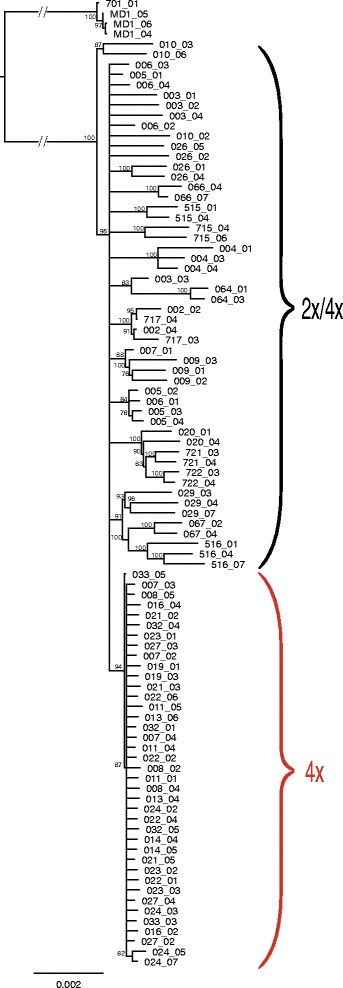
Phylogenetic tree of *M recutita* samples calculated with the neighbor-joining method. The tree derived from the supermatrix consisting of all concatenated sequences and was rooted with the outgroup *M. discoidea* (MD1 and 701). The long branch leading to the outgroup was arbitrarily shortened for clarity. Bootstrap values (bs) of the clades above 75 are indicated close to the nodes

**Fig. 2:**
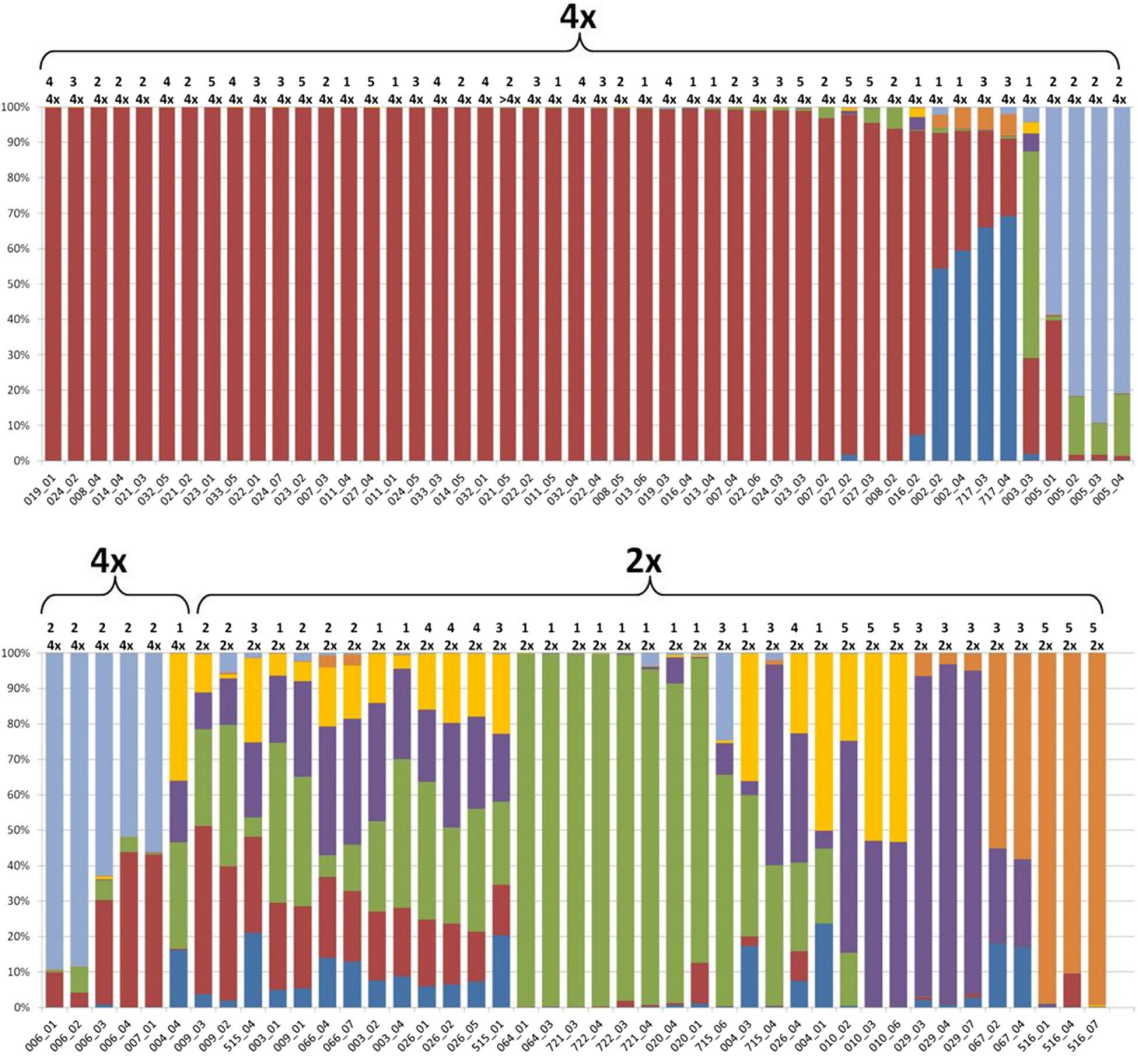
STRUCTURE* analysis (k = 7), with data for geographic origin and ploidy (*both upper rows*). * The barplot is organized according to the ploidy. The genotypes are represented by the vertical bars, whereas the different colours indicate the seven genetic clusters (Additional file 5: Table S1). The top row indicates the geographical origin (*upper*) and ploidy (*lower*) for each sample according to Table 1. The genotypes presented in the order of Table 2 can be found in Additional file 8: Fig. S7

As expected, the 4 genotypes of *M. discoidea* (outgroup) were clearly genetically separated from *M. recutita* (see Fig. 1, Additional file 1: Fig. S1 and Additional file 2: Fig. S2), and were thus excluded from further analyses. Using STRUCTURE, the optimal numbers of clusters (k) that described the population structure of the investigated chamomile were determined as 3 and 7 (Additional file 3: Fig. S3) for the main (Additional file 4: Fig. S4) and more detailed substructures (Fig. 2, Additional file 5: Table S1), respectively. Although the allele frequencies were assumed to be correlated between the origins of chamomile, STRUCTURE was nonetheless additionally run with the setting of independent allele frequencies, which lead to far less population structure differentiation (as expected and described by Falush et al. [25]), but did not significantly differ from the outcome of the analysis with correlated allele frequencies (Additional file 6: Fig. S5). In general, the STRUCTURE analyses demonstrated that the single plants of an origin were genetically similar, with moderate exceptions occurring (Fig. 2, e.g. 005 = ‘Lutea’ or 010 = ‘Argenmilla’).

The plants characterized by 1 or 2 main STRUCTURE clusters, i.e. having one or two STRUCTURE colours > = 9.5% (Fig. 2) were mostly tetraploids (average ploidy of the whole group close to tetraploid: Additional file 7: Fig. S6), whereas all plants except one with 3 to 5 different main STRUCTURE clusters (> = 9.5%) were diploid (Fig. 2, Additional file 7: Fig. S6). Except for one genotype (‘Camoflora’ 004_04) all tetraploids had only 1 or 2 main genetic clusters, whereas diploids showed far more genetic diversity (Fig. 2 and Table 2), although several origins of wild growing chamomile populations were also relatively homogenous (020, 064, 721, 722).

**Table 2:** Ploidy level, flowering time (FT) and quantity of bisaboloids (μg/g) for the investigated plants

Origin	Genotype	ploidy level^a^	FT^b^	Bisabolol oxide B	Bisabolon oxide A	alpha-Bisabolol	Bisabolol oxide A
Manzana1	002_02	4×	230	0	0	400.22	3.17
Manzana1	002_04	4×	232	0	0	735.82	0
Bodegold	003_01	2×	223	6.15	50.3	11.17	119.57
Bodegold	003_02	2×	236	285.35	0	0	190.8
Bodegold	003_03	4×	285	0	0	0	6.2
Bodegold	003_04	2×	246	8.62	27.3	0	145.42
Camoflora	004_01	2×	236	100.82	44.92	0	115.37
Camoflora	004_03	2×	230	12.12	0	0	18.67
Camoflora	004_04	4×	119	35.15	0	0	156.47
Lutea	005_01	4×	239	859.75	0	181.1	21.45
Lutea	005_02	4×	244	23.3	0	506.02	0
Lutea	005_03	4×	239	15.37	0	811.2	0
Lutea	005_04	4×	244	63.8	20.12	9.3	16.8
Zloty Lan	006_01	4×	254	6.3	0	353.15	0
Zloty Lan	006_02	4×	NA	NA	NA	NA	NA
Zloty Lan	006_03	4×	278	490.95	5.12	186.72	0
Zloty Lan	006_04	4×	239	378.15	24.65	0	89
Goral	007_01	4×	232	970.9	0	144.02	23.67
Goral	007_02	4×	252	33.27	852.22	24.62	277.62
Goral	007_03	4×	246	848.27	0	2.47	25.77
Goral	007_04	4×	NA	NA	NA	NA	NA
Bohemia	008_02	4×	243	14.25	106.95	0	0
Bohemia	008_04	4×	257	37.4	43	0	9.3
Bohemia	008_05	4×	236	200.52	351.6	0	0
Promyk	009_01	2×	NA	NA	NA	NA	NA
Promyk	009_02	2×	230	2.19	0	0	0
Promyk	009_03	2×	239	382.27	0	0	7.5
Argenmilla	010_02	2×	78	366.05	176.9	0	323.85
Argenmilla	010_03	2×	78	NA	NA	NA	NA
Argenmilla	010_06	2×	106	487.52	0	0	10.25
trade B + T	011_01	4×	257	527.6	0	0	7.2
trade B + T	011_04	4×	254	69.62	0	0	72.12
trade B + T	011_05	4×	254	239.95	35.12	0	0
trade FStM	013_04	4×	NA	NA	NA	NA	NA
trade FStM	013_06	4×	265	11.45	433.8	0	0
trade PNOS	014_04	4×	254	0	0	0	10.42
trade PNOS	014_05	4×	252	18.45	0	14.95	128.1
trade Kiepenkerl	016_02	4×	236	9.85	0	0	92.85
trade Kiepenkerl	016_04	4×	243	90.87	0	0	59.67
trade Italy 6	019_01	4×	236	14.17	0	0	134.47
trade Italy 6	019_03	4×	265	293.37	0	48.65	3.4
pop England 1	020_01	2×	NA	NA	NA	NA	NA
pop England 1	020_04	2×	NA	NA	NA	NA	NA
trade Garafarm	021_02	4×	257	17.32	0	0	79.57
trade Garafarm	021_03	4×	252	664.47	13.35	0	0
trade Garafarm	021_05	> 4× ^c^	236	127.75	0	0	133.8
Margaritar	022_01	4×	246	478.2	10.55	3.92	7.2
Margaritar	022_02	4×	243	165.32	49.22	35.1	0
Margaritar	022_04	4×	246	159.25	2.2	0.32	0
Margaritar	022_06	4×	246	13.9	0	0	0
trade Agbina	023_01	4×	239	17.62	0	0	122.1
trade Agbina	023_02	4×	236	185.4	39.95	0	174.15
trade Agbina	023_03	4×	230	35.45	48.32	0	0
Lazur	024_02	4×	243	20.77	0	0	218.77
Lazur	024_03	4×	247	650.7	0	0	14.05
Lazur	024_05	4×	239	178.1	0	0	131.85
Lazur	024_07	4×	252	0	0	0.42	58.27
Germania	026_01	2×	246	74.32	0	0	572.77
Germania	026_02	2×	NA	NA	NA	NA	NA
Germania	026_04	2×	259	12.72	31.22	0	162.07
Germania	026_05	2×	236	922.9	0	0	32.4
trade USA	027_02	4×	246	5.86	67.8	0	74.77
trade USA	027_03	4×	272	NA	NA	NA	NA
trade USA	027_04	4×	252	133.95	147.7	0	0
pop Croatia	029_03	2×	78	0.5	0	21.02	0
pop Croatia	029_04	2×	83	3.5	0	0	56.75
pop Croatia	029_07	2×	218	129.57	0	0	2.4
trade Italy 5	032_01	4×	236	131.27	0	0	0
trade Italy 5	032_04	4×	239	152.35	0	0	164.05
trade Italy 5	032_05	4×	243	97.62	0	0	134.77
trade Italy 4	033_03	4×	239	732.62	28.3	3.52	13.47
trade Italy 4	033_05	4×	252	854.7	0	0	11.65
pop Germany 1	064_01	2×	236	31.52	98.65	2.92	124.45
pop Germany 1	064_03	2×	260	78.37	101.5	2.35	242.37
Pohorelicky Velkokvety	066_04	2×	239	0	81.32	0	294.95
Pohorelicky Velkokvety	066_07	2×	239	77.79	65.62	0	209.92
pop Bulgaria 1	067_02	2×	NA	NA	NA	NA	NA
pop Bulgaria 1	067_04	2×	NA	NA	NA	NA	NA
Krajovy	515_01	2×	239	186.65	0	0	262.25
Krajovy	515_04	2×	NA	NA	NA	NA	NA
pop North Korea 1	516_01	2×	NA	NA	NA	NA	NA
pop North Korea 1	516_04	2×	NA	NA	NA	NA	NA
pop North Korea 1	516_07	2×	146	0	337.35	0	0
Bona	715_04	2×	106	0	0	19.87	0
Bona	715_06	2×	NA	NA	NA	NA	NA
Manzana2	717_03	4×	236	0	0	165.1	0
Manzana2	717_04	4×	239	0	0	437.27	0
pop England 2	721_03	2×	239	17.65	0	0	192.92
pop England 2	721_04	2×	232	105.25	0	1.47	144.27
pop England 3	722_03	2×	244	52.32	0	0	0.85
pop England 3	722_04	2×	225	143.7	9.27	0	157.72
M. discoidea 1	701_01		78	0	41.07	0	0
M. discoidea 2	MD1_04		78	0	79.35	4.92	1
M. discoidea 2	MD1_05		78	0	29.75	0	0
M. discoidea 2	MD1_06		78	0	29.87	1.15	0

^a^measured between the 26th of August 2014 and the 03rd of September 2014

^b^measured as days after sowing (DAS) to the start of flowering: NA = no flowering (plant dead before flowering); 78–200 DAS: short day conditions; 201–260 DAS: long day conditions

^c^ploidy slightly higher than tetraploid level (7%), grouped within the tetraploid plants

In particular, the 39 genotypes from 14 tetraploid origins (USA and throughout Europe) were genetically similar and homogenous, forming one large group (see Fig. 2, Additional file 8: Fig. S7: red coloured bars, Fig. 1: parenthesis “4×”, Additional file 1: Fig. S1: red coloured dots and Additional file 9: Fig. S8a). Furthermore, 9 tetraploid genotypes (‘Lutea’ = 005, ‘Zloty Lan’ = 006 and one genotype ‘Goral’ = 007_01) and all four plants ‘Manzana’ (both origins 002 and 717) formed two additional groups. ‘Manzana’, like several other origins (e.g. an accession from North Korea (516), ‘Camoflora’ (004) and a genebank accession from Bulgaria (067)), was identified as being genetically different from most of the origins (Fig. 2). No clear connection between the genetic structure (clusters) and the geographic origin was apparent, i. e. no geographic differentiation could be identified (Fig. 2 and Additional file 9: Fig. S8b), even though the origins from Argentina (010) and North Korea (516) indeed seem to be a little bit more distinct (Additional file 9: Fig. S8b).

### Average flowering time for diploid chamomile is shorter than for tetraploids

**Fig. 3:**
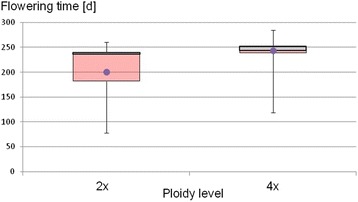
Flowering time [d] for 27 diploid (2×) and 51 tetraploid (4×) *M. recutita* plants. Box-whisker-plots of days after sowing (DAS) when flowering started, for diploid and tetraploid plants. The quartiles 2 and 3 are drawn with the median in between them. The whiskers indicate the maximum and minimum values, whereas the dot in the middle represents the arithmetic mean (Additional file 10: Table S2)

Diploid chamomile plants flowered significantly earlier than tetraploid ones (Fig. 3, Additional file 10: Table S2: average flowering time 201 d vs. 244 d, t-test: *P* = 4.20 × 10^−5^), and most of the plants (70 out of 78) flowered under long-day conditions, that is 200 DAS. Tetraploid plants showed a narrower range of FT than diploid ones (Fig. 3). In total only 8 genotypes and the 4 *M. discoidea* plants as outgroup flowered under short-day conditions (Table 2: all three from Argenmilla (010), two out of three from “population Croatia” (029_03, 029_04), and one from each origin ‘Bona’ (715_04), ‘Camoflora’ (04_04), and North-Korea (516_07)). All genotypes flowering under short-day conditions were diploid except for one tetraploid genotype (“04_04”) from the variety ‘Camoflora’. A PCoA-analysis revealed for most of these plants (except 715_04 and 04_04) clear differences to the later flowering ones (Additional file 9: Fig. S8c).

### The content of the bisaboloids was highly variable between the genotypes

**Fig. 4:**
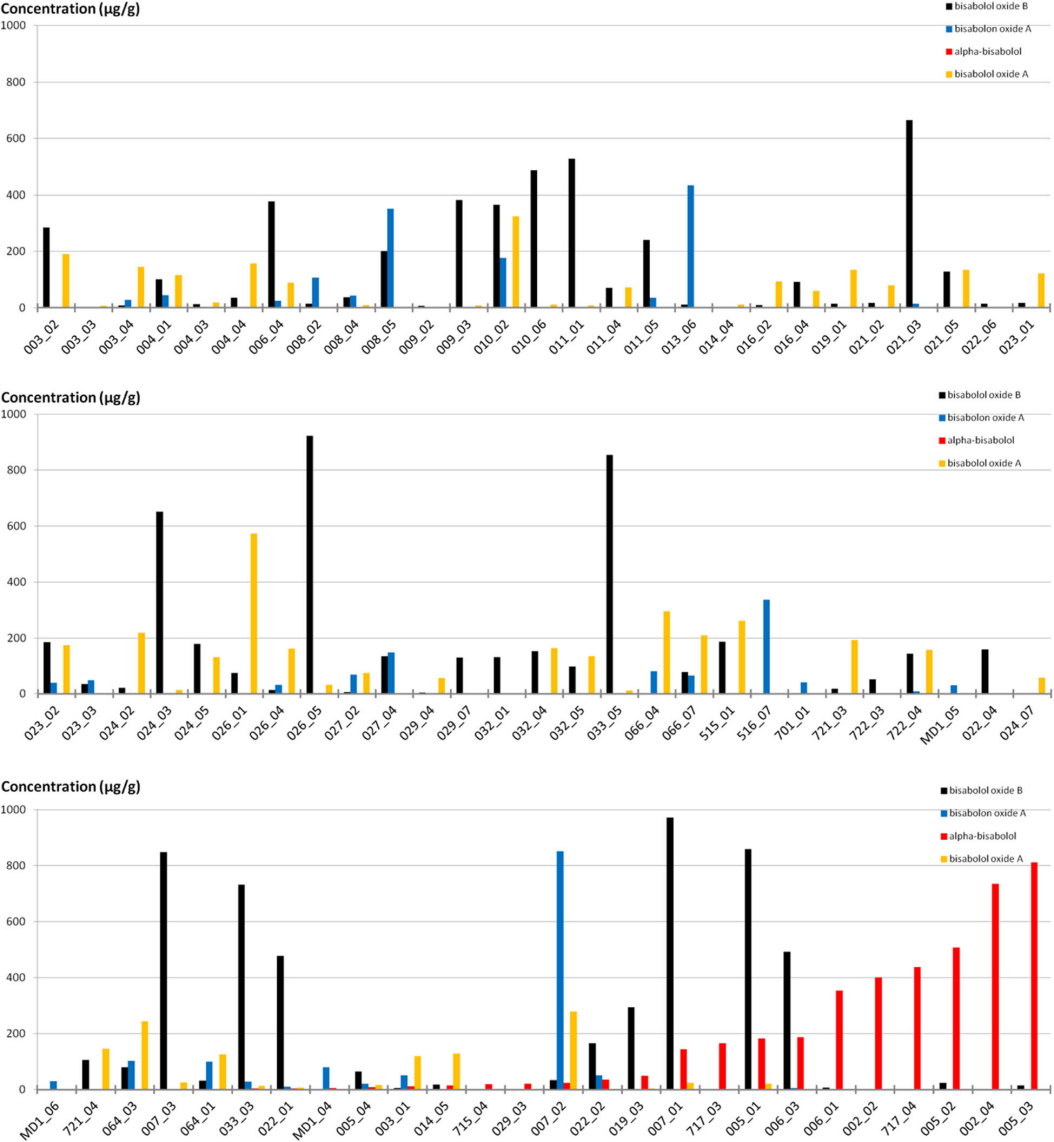
Content of bisaboloids [μg/g] in the pooled flower heads, genotypes ordered by alpha-bisabolol content

Large differences in the content of single bisaboloids were measured between the genotypes and origins, with single genotypes tending to be rich in only one of the four bisaboloids, i.e. plants showing a high content of alpha-bisabolol often had a low content of bisabolol oxide A and bisabolon oxide A (Fig. 4, Table 2). The plants from the outgroup *M. discoidea* had low contents of bisaboloids, only bisabolon oxide A was present to some extent (Fig. 4).

In total, 52 plants out of 80 showed no detectable content of alpha-bisabolol. The plants with high levels of the medicinal important substance bisabolol Fig. 4) were the 4 genotypes from ‘Manzana’ (002_02, 002_04, 717_03, 717_04) and the genetically defined group (see above and Fig. 2) consisting of ‘Camoflora’, ‘Lutea’ and one genotype from ‘Goral’ (007_01). The alpha-bisabolol rich genotypes were characterized by two main STRUCTURE clusters each (> = 9.5% share of each genetic cluster, compare Table 2 and Fig. 2).

### Genome-wide association study (GWAS) identified significantly associated SNP-markers for the traits flowering time and alpha-bisabolol

**Fig. 5:**
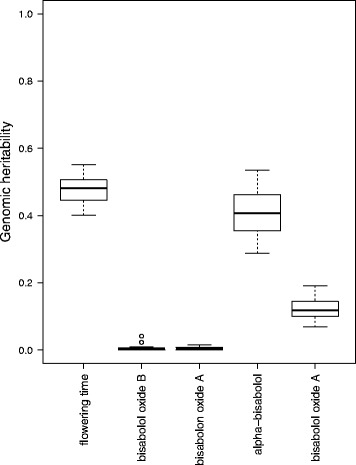
Genomic heritability* for flowering time and content of the four bisaboloids. *The genomic heritability is given in a range between 0 and 1 (y-axis), and estimates how much of the observed phenotypic variation can be explained genetically. In the box-whisker-plot the quartiles 2 and 3 are drawn, with the median in between them. The whiskers indicate the maximum and minimum values (Additional file 11: Table S3)

The estimated genomic heritabilities for the traits flowering time and content of alpha-bisabolol were above 0.4 (0.48 and 0.40 respectively, Fig. 5, Additional file 11: Table S3), while that for the content of bisabololoxide B, bisabolone oxide A and bisabololoxide A were clearly lower (0.01; 0.01; 0.14, Fig. 5, Additional file 11: Table S3). Thus, genome-wide association studies were performed for flowering time and alpha-bisabolol only, focusing on the two traits for which a high portion of the phenotypic variability can be explained genetically.

GWAS resulted in the identification of 9 SNPs located in 5 different sequences for flowering time (FT) and 72 SNPs located in 60 different sequences for alpha-bisabolol content, all of which were above the defined significance threshold 0.01 under Bonferroni correction (Additional file 12: Table S4). Three sequences (2445, 3029, 4585) harboured more than one SNP associated with FT, and ten sequences (256, 1379, 1998, 2208, 2380, 2489, 5681, 6623, 6440, 6670) more than one SNP associated with alpha-bisabolol content.

#### Flowering time (FT)

The BLAST alignment [40, 41] of the 5 sequences harbouring significantly associated SNPs (Additional file 12: Table S4) against the NCBI nucleotide collection of flowering plants identified 2 sequences with significant hits to potential candidate genes (BLAST-score of > = 80 with a sequence identity of > = 80% and an E-value <1 × 10^−13^) in multiple plant species. Both sequences harboured more than one significantly associated SNP: (1) “seq. 2445” with 2 SNPs: xyloglucan endotransglucosylase/hydrolase protein with significant alignments to 30 plant species; and (2) “seq. 4585” with 3 SNPs: uncharacterized protein with significant alignments to 9 plant species, and protein kinase / salt-induced ABC1 kinase below the BLAST aligment results threshold in 2 plant species (Additional file 13: Table S5). Xyloglucan endotransglucosylase/hydrolase is described to be involved in self-pollination in *Arabidopsis thaliana* [47].

BLAST aligment (megablast) of the FT associated sequences to the sunflower genome [46] yielded hits for 4 of the 5 sequences on different chromosomes (multiple hits on 6 chromosomes for “2445”, single hits all in different chromosomes for “441”, “3029” and “4585”).

#### Alpha-bisabolol content

Out of the 60 sequences (contigs) associated with alpha-bisabolol content in our study (Additional file 12: Table S4), 31 could be aligned by BLAST against the NCBI nucleotide collection of flowering plants, of which 13 sequences achieved matches above the selected threshold (listed in Additional file 13: Table S5 together with the suggested functional role of the putative gene: BLAST-score of > = 80 with a sequence identity of > = 75% and an E-value <1.33 × 10^−13^). For 12 of these 13 sequences specific gene products were described in multiple plant species (Additional file 13: Table S5).

Whereas 8 (sequences 3218, 3223, 3249, 4194, 6598, 6694) of the 12 sequences are involved in synthesis or regulation within universal pathways and associated ubiquitous proteins in many eukaryotes or plants (Additional file 13: Table S5), 4 sequences are described to play a role in plant biotic and abiotic stress response. Interestingly, one sequence (3223) is described to be involved both in basic function and in abiotic stress response ([48]: armadillo/beta-catenin repeat family proteins). Ascorbate peroxidase (722) responses are directly involved in the protection of plant cells against adverse environmental conditions [49], Glutamate receptor-like channels (1139) in plants are discussed to fulfill a function in response to an attack from pests and pathogens [50], and the chaperone protein ClpB1 (5721) is described to be a heat shock protein required for acclimation to high temperatures [51]. The last sequence (1998) is predicted to encode a nuclear fusion defective 4-like protein, which is described to be required for karyogamy during female gametophyte development and fertilization in *Arabidopsis thaliana* [52].

The BLAST aligment (megablast) of the 60 sequences associated with alpha-bisabolol content against the sunflower genome [46] yielded 35 matches above the threshold for the E-value of 10^−3^. 12 of the 13 sequences from the alignment to different plant genomes (Additional file 13: Table S5, except the microsatellite sequence “1379”) were above the threshold value for the alignment against sunflower genome.

The 35 sequences with E-value above the threshold, were aligned to all 17 chromosomes of the sunflower genome, with minimum of 1 (chromosome 17) and maximum of 7 to 8 (chromosomes 8, 10 and 13) sequences per chromosome (Additional file 14: Table S6). Among these 35 sequences, 14 were aligned to only 1 sunflower chromosome, 10 to 2 different sunflower chromosomes, and the remaining 11 to multiple chromosomes, with sequences “4194” and “5751” both being aligned to 6 chromosomes. Ten sequences were aligned to multiple loci on the same chromosome. Tblastx gave more hits against the sunflower genome, but with higher E-value and only consisting of more multiple hits against the same chromosomes.

The sequences for which specific gene products in multiple plant species were identified (see above) were not concentrated on any of the sunflower chromosomes (Additional file 14: Table S6: sequence name written bold). Based on these data, no clustering of these 35 highly alpha-bisabolol associated sequences to single sunflower chromosomes could be observed.

Mapping by Blast2GO of the BLAST results to retrieve gene ontology terms for FT and alpha-bisabolol content achieved no matches. Thus, no functional annotation could be done.

## Discussion

### Rare autopolyploidization events are a potential cause for low genetic diversity of the cultivated tetraploid chamomile

Tetraploid chamomile varieties were generated a very few times artificially by polyploidisation of diploid plants, as evidenced by the STRUCTURE analyses (Fig. 2). The large, genetically homogenous group of 14 tetraploid orgins, obtained from all over Europe, is likely to originate from the same polyploidised diploid genetic background, i.e. similar parental material, with minor genetic differences arising via selection and introgression during breeding. These data indicate that most of the high-performing tetraploid chamomile origins are genetically highly similar, and justify the demand to further broaden the diversity by including different germplasm in the future breeding process. Two smaller, genetically different groups of tetraploid origins with “extended” genetic background include plants with the highest alpha-bisabolol content (see below): (1) The tetraploid variety ‘Manzana’, which was generated from diploid ‘Degumille’ [53] and (2) the group containing ‘Lutea’, ‘Zloty Lan’ and partly ‘Goral’.

Two tetraploid genotypes (003_03 from ‘Bodegold’, 004_04 from ‘Camoflora’) were genetically diverse, each consisting of several STRUCTURE-clusters, although both were similar in “STRUCTURE fingerprints” to their corresponding diploid genotypes. Considering ‘Bodegold’, registered in 1962 as a tetraploid variety [54], which consisted of di- and tetraploid plants, we hypothesize that contamination with closely related wild growing diploid chamomile through seeds or (unreduced) pollen has led to variable ploidy [19]. Contrarily, ‘Camoflora’ (a diploid variety) might have experienced fertilization with both unreduced male and female gametes or unreduced egg cells crossed with cultivated tetraploid chamomile, which both could have led to the detection of tetraploid genotypes in our study. However, frequent gene flow between di- and tetraploid chamomile is not expected due to the different ploidy of the gametes, which would typically lead to highly sterile triploid F_1_-progeny [55, 19]. Consequently, an increase in the genetic diversity of tetraploid chamomile origins by crosses with diploids is not likely to happen spontaneously, and hence the inclusion of different new origins in breeding programmes would likely be advantageous.

### Geographic origin showed no congruence with genetic structure

In our analysis, there was no clear congruence between the genetic structure (clusters) and geographic origin (Fig. 2 and Additional file 9: Fig. S8b), i.e. no clustering due to geographic origin could be found. Even though the origins from Argentina (010) and North Korea (516) indeed seem to be genetically distinct from the European ones (Additional file 9: Fig. S8b), the samples from Russia (23), Egypt (26) and USA (27) showed strong similarity to diverse European origins. An explanation could be that regular exchange of plant material between the regions took place for this mainly outcrossing species, and that in our panel of plants were mainly cultivated chamomile origins with good agricultural performance. Franke and Schilcher [9] discriminated the origin of chamomile flowers by their chemotype, but only from wild and not cultivated collections. For the crop plant *Brassica rapa*, Tanhuanpää et al. [56] analysed 61 accessions with SNP markers and found grouping corresponding to morphotype and flowering habit, but not to geographic origin. Their results are comparable to our data, indicating the limited value of the geographic origin alone (without correlation to other traits) to genetically structure cultivated outcrossing plants for which the exchange of genetic material is not inhibited. Consequently, only with numerous wild collected samples from the natural regions of origin (e.g. Iran) specific geographic footprints in the genetic patterns might be revealed.

### Flowering started earlier for diploid than for tetraploid chamomile plants

Diploid plants flowered in average significantly earlier than tetraploid ones (Fig. 3, Additional file 10: Table S2), as has been shown for *Chamerion angustifolium* [57, 58]. It is hypothesized that (auto-) tetraploid plants develop slower and flower later due to the longer time necessary for replication of a doubled genome [59, 60], which consequently may lead to slowing of metabolism and growth rates in polyploids [61, 62]. Since tetraploid chamomile is autotetraploid, possible effects of allopolyploidy like allelic interactions or epigenetic modifications of homoeologous loci leading to nonadditive gene regulation [63, 64], are not considered.

The high genomic heritability observed for the flowering time in our study (Fig. 5) additionally indicates a strong genetic influence on the flowering time in chamomile, as has been described for *Arabidopsis* and *Brassica*. Mayfield et al. [64] hypothesized that late flowering in these genera is a dominant trait, as flowering time probably depends on the expression of a repressor gene. Thus, late flowering in tetraploids compared to diploids could be additionally influenced by higher chances for dominant alleles to be present. Bao et al. [65] identified factors regulating flowering and endopolyploidization in *Arabidopsis* and suggest an involvement of cell cycle control in the timing of reproductive transition.

### Identification of the flowering time (FT) associated sequences in chamomile can enhance breeding

The 5 sequences containing the SNPs significantly associated with flowering time (Additional file 12: Table S4) may serve as a starting point to develop molecular markers to be used in marker assisted selection (MAS) for FT in chamomile. Early flowering (=early harvesting) could help circumvent negative effects on yield due to summer drought, whereas late flowering might be of advantage in cold regions with late frost, especially when sowing was already done in autumn. Additionally to the effect of the ploidy on FT, molecular markers could facilitate the breeding for early or late flowering varieties by selecting genotypes with genetic factors for these traits.

The BLAST alignment demonstrated that the orthologue of one of these 5 sequences (“2445”; Additional file 12: Table S4) codes for protein connected to flowering (i.e. self-pollination) in *A. thaliana* [47]. Further investigations would be required to unravel the function of the identified sequences in chamomile.

### High alpha-bisabolol content detected in tetraploid plants from two genetically defined groups indicates its limited occurence in cultivated chamomile

Only 9 chamomile genotypes (origins ‘Manzana’, ‘Lutea’, ‘Goral’ and ‘Zloty Lan’) from 2 STRUCTURE groups (Fig. 2) showed a high content of alpha-bisabolol, as has been described for ‘Manzana’, ‘Lutea’, and ‘Goral’ [2]. Here, only one out of three analysed ‘Goral’ genotypes showed a high bisabolol level, while two of three ‘Zloty Lan’ genotypes were alpha-bisabolol-rich with the third genotype being rich in alpha-bisabolol oxide B, in contrast to the data of Franke and Schilcher [2] and Das [7]. Our data for ‘Lazur’ also differ from the data of Franke and Schilcher [2] as we detected high levels of bisabolol oxide A and B but no bisabolon. These differences could be explained by genotypic variability in the polymorphic varieties of this outcrossing species, potentially increased by introgression with other chamomile populations during cultivation. This potential variability should be taken into account when maintaining a variety characterized by high-levels of specific compounds serving as medicinal drug, as well as when breeding new varieties.

The pharmaceutically important sesquiterpene alpha-bisabolol was detected in 28 of the analysed plants. It was previously suggested that an oxidative biosynthetic pathway converts the alpha-bisabolol to bisabolol oxide B or to bisabolol oxide A, and from the latter further to bisabolone oxide A [66, 67].

The 9 alpha-bisabolol rich genotypes belonged to 4 varieties (see above) from 2 different tetraploid groups, both possessing 2 main genetic clusters as defined by STRUCTURE (compare Table 2 and Fig. 2). However, a general conclusion that heterotic effects lead to the higher content of alpha-bisabolol cannot be deduced from our data (Fig. 4 and Fig. 2), as many origins with several main genetic clusters each had no detectable alpha-bisabolol content. Since only 2 tetraploid groups showed plants with high alpha-bisabolol content in our study, their membership in two main genetic clusters could also be a random effect explained by a breeding process of choosing different genetic materials as parents, but not necessarily leading to significant heterotic effects. This view is also supported by the analysis of the average heterozygosity. Some alpha-bisabolol rich genotypes displayed an elevated level of heterozygosity over the whole DNA-sequences, but more genotypes with the same level of heterozygosity contained no alpha-bisabolol (Additional file 15: Fig. S9, Additional file 16: Table S7). Wagner et al. [16] describe the inheritance of the ability to produce alpha-bisabolol as monogenic, which does not support the involvement of a heterosis effect in its content. The gene most likely responsible for alpha-bisabolol formation is the alpha-bisabolol synthase McTPS2 identified in chamomile [68]. The expression of such a terpene synthase was often found to be dominant in many plants including maize [69, 70]. In a genome wide association study of maize, several SNPs with significance for terpene production were located in terpene synthase genes [71]. However, in chamomile, none of the SNPs associated with alpha- bisabolol content were located in the sequences of alpha-bisabolol synthase or other genes with an apparent involvement in terpene biosynthesis [21]. Some of the SNPs controlling alpha-bisabolol biosynthesis may affect regulatory factors that also cause a positive introgression/heterosis effect.

It cannot be concluded that tetraploidy leads to an enhanced potential for elevated levels of alpha-bisabolol, since only some tetraploids had high levels of it, and for ‘Manzana’ the diploid starting material (‘Degumille’: [53]) is already described to contain high levels of alpha-bisabolol.

### Connection between high alpha-bisabolol content with genes for plant defense and stress response

Thirteen out of the 60 sequences harbouring the 72 SNPs significantly associated with alpha-bisabolol content could be annotated, 9 of which had orthologues in multiple plant species, of which 4 are described with roles in plant biotic and abiotic stress response (Additional file 13: Table S5).

The association of genes for plant defense and stress response with alpha-bisabolol content is consistent with the antifungal and antibacterial effects of alpha-bisabolol and its oxides [10, 72], which are part of their medical value. Secondary metabolites often function in plant defense and stress response, and in particular, terpenoids (a class of substances to which the bisaboloids belong) play vital roles in plant defense [73]. Furthermore, synthesis and accumulation of natural products (i.e. secondary metabolites) is enhanced in drought-stressed plants, or by application of signal transducers [74, 75]. Hence, we hypothesize that moderate stress might enhance the production and thus concentration of alpha-bisabolol in chamomile, and yield as long as the overall growth of the plant is not hampered.

## Conclusion

Our analyses of high quality SNPs enabled us to identify genetic clustering in chamomile, with some genotypes belonging mainly to one genetic cluster, while others demonstrate evidence for introgression beween different genetic clusters. Tetraploid varieties tended to be less diverse than the diploid ones. Several origins were identified as being genetically different from most other origins, suggesting their possible use for exploiting heterosis. We identified several genotypes with high levels of the medicinal important substance alpha-bisabolol. Single genotypes tended to be enriched in only one of the four bisaboloids. GWAS identified highly significant (*P* < 0.01) SNPs for flowering time (9) and alpha-bisabolol content (71). The data from GWAS pave the way for future research towards the identification of marker trait associations, their underlying genetics and their use in marker assisted selection (MAS) for practical breeding in German chamomile.

## Additional files

Additional file 1: Figure S1. PCoA analysis of the 95 samples reveals the outgroup *M. discoidea* to be clearly distinct from *M. recutita.* Colours: green: outgroup from *M. discoidea* (701_01, MD1_04, MD1_05, MD1_06); red: homogenous group of 14 tetraploid origins (007_02, 007_03, 007_04, 008_02, 008_04, 008_05, 011_01, 011_04, 011_05, 013_04, 013_06, 014_04, 014_05, 016_02, 016_04, 019_01, 019_03, 021_02, 021_03, 021_05, 022_01, 022_02, 022_04, 022_06, 023_01, 023_02, 023_03, 024_02, 024_03, 024_05, 024_07, 027_02, 027_03, 027_04, 032_01, 032_04, 032_05, 033_03, 033_05); blue: rather diverse group consisting of all remaining samples*.* Principal coordinate analysis (PCoA, Gower 1966) based on one Euclidean distance matrix termed modified Rogers’ distance (mRD, Wright 1978; Goodman and Stuber 1983; Reif et al. 2005) as another presentation of population structure. The specific algorithm was illustrated in Reif et al. 2005. Corresponding programming is executed in R (R Core Team 2013). References: Goodman M M, Stuber C W. Races of maize. 6: Isozyme variation among races of maize in Bolivia[R]. 1983. Gower JC (1966) Some distance properties of latent root and vector methods used in multivariate analysis. Biometrika. 53(3–4):325–338. R Core Team (2013). R: A language and environment for statistical computing. R Foundation for Statistical Computing, Vienna, Austria. ISBN 3–900,051–07-0, URL http://www.R-project.org/. Reif J C, Melchinger A E, Frisch M. Genetical and mathematical properties of similarity and dissimilarity coefficients applied in plant breeding and seed bank management[J]. Crop Science, 2005, 45(1): 1–7. Wright, S. 1978. Evolution and genetics of populations. Vol. IV. The Univ. of Chicago Press. (DOCX 62 kb)

Additional file 2: Figure S2. STRUCTURE* analysis (K = 4) including outgroup *M. discoidea* (last 4 samples, blue) reveals strict genetic separation from *M. recutita*. * The genotypes are represented by the vertical bars, whereas the different colours indicate the four genetic clusters. (DOCX 48 kb)

Additional file 3: Figure S3. Calculation of Delta K according to Evanno et al. (2005) by structure harvester for K = 1 to 15 (Earl and von Holdt 2012; K = number of clusters) determines values of 3 and 7 to fit the data best (DOCX 25 kb)

Additional file 4: Figure S4. STRUCTURE analysis assuming 3 clusters (K = 3). * The genotypes are represented by the vertical bars, whereas the different colours indicate the three genetic clusters (Additional file 5: Table S1). (DOCX 48 kb)

Additional file 5: Table S1. The different genetic clusters (K = 7) from the STRUCTURE analysis for the chamomile genotypes. (DOCX 18 kb)

Additional file 6: Figure S5. STRUCTURE* analysis with allele frequencies assumed to be independent among populations and for 7 clusters (K) leads to low population structure differentiation. * The genotypes are represented by the vertical bars, whereas the different colours indicate the seven genetic clusters. (DOCX 45 kb)

Additional file 7: Figure S6. Average ploidy level for the groups of plants organized according to their number of main STRUCTURE-clusters (> = 9.5%). (DOCX 35 kb)

Additional file 8: Figure S7. STRUCTURE* analysis assuming 7 clusters (K = 7), organized according to Table 2. * The genotypes are represented by the vertical bars and the different origins are separated by vertical black lines, whereas the different colours indicate the seven genetic clusters (Additional file 5: Table S1). The top row indicates the geographical origin (upper) and ploidy (lower) for each sample according to Table 1 (DOCX 516 kb)

Additional file 9: Figure S8. PCoA analysis of the 91 samples *M. recutita* coloured according to (a) ploidy, (b) geographic origin, (c) flowering time. (a) ploidy. (b) geographic origin. (c) flowering time. PCoA performed as described in Additional file 1: Fig. S1. (a): red: diploid, green: tetraploid (b) code for geographic origin see Table 1 (c) start flowering from days after sowing (DAS): 1 (78 DAS - 107 DAS), 2 (108 DAS - 200 DAS), 3 (201 DAS - 260 DAS), 4 (261 DAS - 298 DAS), 1–2: short day conditions; 3–4: long day conditions. (DOCX 431 kb)

Additional file 10: Table S2. Flowering time for diploid and tetraploid chamomile (DOCX 11 kb)

Additional file 11: Table S3. Genomic heritability: result from analysis 20 times with 5-fold cross validation (DOCX 13 kb)

Additional file 12: Table S4. Significant SNPs for the traits flowering time (ft, 9 SNPs) and alpha-bisabolol content (bis, 72 SNPs)*. * The SNPs for the traits flowering time (ft, 9 SNPs) and alpha-bisabolol content (bis, 72 SNPs) are contained in 5 and 60 different DNA-sequences, respectively. The sequences in the table are organized according to the *p*-value. ** Red letters in the sequence indicate the SNPs identified for this sequence, whereas bold red letters indicate the actual SNP for which the data are given in the corresponding line and for which the other alleles are listed in the fourth column. The single-letter IUPAC nucleotide code is used. (DOCX 23 kb)

Additional file 13: Table S5. BLAST alignment results* for 5 and 13 sequences harbouring SNPs significantly associated with flowering time (FT) and alpha-bisabolol, respectively. * BLAST alignment results threshold: BLAST-score of > = 80 with a sequence identity of > = 80%/75% and an E-value <1.33 × 10^−13^; (in brackets): results below the threshold (minimum BLAST-score of >55 with a sequence identity of > = 75% and an E-value <1 × 10^−4^. The blastn algorithm was used for the analysis of the data, since the results obtained from tblastx were similar to the ones from blastn. Tblastx achieved not more hits above the significance threshold than blastn at the NCBI database. ** A more general annotation approach, an HMM (hidden Markov model) search through the Protein families database, did also not yield any results for sequence 6153. *** tblastx. The BLAST alignment of the 5 sequences harbouring significantly associated SNPs to FT (Additional file 12: Table S4) against the NCBI nucleotide collection of flowering plants identified 2 sequences with significant hits to potential candidate genes in multiple plant species (BLAST-score threshold see above, with a sequence identity of > = 80%). Below the significance threshold, for 3 sequences an alignment to plant species could be done, but not for sequence 6153. (1) Jankowsky E. RNA Helicases at work: binding and rearranging. *Trends in biochemical sciences*. 2011;36(1):19–29. doi:10.1016/j.tibs.2010.07.008. (2) Kurasawa K, Matsui A, Yokoyama R, Kuriyama T, Yoshizumi T, Matsui M, Suwabe K, Watanabe M, Nishitani K. The AtXTH28 gene, a xyloglucan endotransglucosylase/hydrolase, is involved in automatic self-pollination in *Arabidopsis thaliana*. Plant Cell Physiol. 2009; 50(2):413–22. doi: 10.1093/pcp/pcp003. From the above 13 alpha-bisabolol associated sequences, one has been described as microsatellite sequence (1379). For the remaining 12 sequences, specific gene products were described in multiple plant species: “722” ascorbate peroxidase 2-like protein, 3 species; “1139” glutamate receptor, 2 species; “1732” predicted: probable carboxylesterase 6, 5 species; “1998” predicted: nuclear fusion defective 4-like (NFD4; LOC109841189) protein, 2 species; “3218” ammonium transporter 3 member 1-like, 2 species;, “3223” U-box domain-containing protein 13 and armadillo/beta-catenin repeat family protein, 3 species; “3249” probable pre-mRNA-splicing factor ATP-dependent RNA helicase, 23 species; “4194” mostly asparagine synthetase, 60 species; “5721” chaperone protein ClpB1/ heat shock protein, 42 species; “6386” lipase, 1 species; “6598” malate dehydrogenase, chloroplastic-like, 19 species; “6654”, mainly reticulon-like protein B2, 24 species. (3) Portereiko MF, Sandaklie-Nikolova L, Lloyd A, Dever CA, Otsuga D, Drews GN NUCLEAR FUSION DEFECTIVE1 encodes the Arabidopsis RPL21M protein and is required for karyogamy during female gametophyte development and fertilization. Plant Physiol. 2006; 141:957–965. (4) Caverzan A, Passaia G, Rosa SB, Ribeiro CW, Lazzarotto F, Margis-Pinheiro M. Plant responses to stresses: Role of ascorbate peroxidase in the antioxidant protection. Genetics and Molecular Biology. 2012;35 (4 Suppl):1011–1019. (5) Forde BG, Roberts MR. Glutamate receptor-like channels in plants: a role as amino acid sensors in plant defence? *F1000Prime Reports*. 2014;6:37. doi:10.12703/P6-37. (6) Sharma M, Pandey A, Pandey GK. β-catenin in plants and animals: common players but different pathways. *Frontiers in Plant Science*. 2014;5:143. doi:10.3389/fpls.2014.00143. (7) Lee U, Rioflorido I, Hong SW, Larkindale J, Waters ER, Vierling E.: 2007. The Arabidopsis ClpB/Hsp100 family of proteins: chaperones for stress and chloroplast development. Plant J. Jan;49(1):115–27 (DOCX 24 kb)

Additional file 14: Table S6. BLAST aligment (megablast) against the sunflower genome (https://www.heliagene.org/HanXRQ-SUNRISE/: *Helianthus annuus* XRQ genome assembly. The chamomile sequence names in bold letters were aligned by BLAST against NCBI nucleotide collection of flowering plants, and for them gene products were described in multiple plant species above the alignment threshold (Additional file 13: Table S5). (DOCX 23 kb)

Additional file 15: Figure S9. Alpha-bisabolol in relation to the average heterozygosity for all polymorphic SNPs for the chamomile genotypes. The high alpha-bisabolol rich genotypes displayed an elevated level of heterozygosity, but more genotypes with the same level of heterozygosity contained no alpha-bisabolol (R2 = 0.056). The underlying data are listed in Additional file 16: Table S7. The two datasets used: (1) demultiplexed fasta-file of the barcoded reads for each genotype and. (2) the matrix for the filtered 6495 SNPs will be made publicly available after acceptance via e!DAL (http://edal.ipk-gatersleben.de) with a proprietary DOI, and during the revision process sent on request by the corresponding author. (DOCX 55 kb)

Additional file 16: Table S7. Number of SNPs showing heterozygosity and alpha-bisabolol content for the single chamomile genotypes. ^**1**^ In relation to 44,468 variable sites (polymorphic SNPs) mined by the pyRAD pipeline before filtering. ^**2**^ all values: × 10^6 (DOCX 16 kb)^
