# Supplementary material for: Use of genotyping-by-sequencing to determine the genetic structure in the medicinal plant chamomile, and to identify flowering time and alpha-bisabolol associated SNP-loci by genome-wide association mapping
Source: BMC Genomics. 2017 Aug 10;18:599. doi: 10.1186/s12864-017-3991-0 (PMC5553732; doi:10.1186/s12864-017-3991-0)
Supplement: Supplementary file 1 — PCoA analysis of the 95 samples reveals the outgroup M. discoidea to be clearly distinct from M. recutita. Colours: green: outgroup from M. discoidea (701_01, MD1_04, MD1_05, MD1_06); red: homogenous group of 14 tetraploid origins (007_02, 007_03, 007_04, 008_02, 008_04, 008_05, 011_01, 011_04, 011_05, 013_04, 013_06, 014_04, 014_05, 016_02, 016_04, 019_01, 019_03, 021_02, 021_03, 021_05, 022_01, 022_02, 022_04, 022_06, 023_01, 023_02, 023_03, 024_02, 024_03, 024_05, 024_07, 027_02, 027_03, 027_04, 032_01, 032_04, 032_05, 033_03, 033_05); blue: rather diverse group consisting of all remaining samples. Principal coordinate analysis (PCoA, Gower 1966) based on one Euclidean distance matrix termed modified Rogers’ distance (mRD, Wright 1978; Goodman and Stuber 1983; Reif et al. 2005) as another presentation of population structure. The specific algorithm was illustrated in Reif et al. 2005. Corresponding programming is executed in R (R Core Team 2013). References: Goodman M M, Stuber C W. Races of maize. 6: Isozyme variation among races of maize in Bolivia[R]. 1983. Gower JC (1966) Some distance properties of latent root and vector methods used in multivariate analysis. Biometrika. 53(3–4):325–338. R Core Team (2013). R: A language and environment for statistical computing. R Foundation for Statistical Computing, Vienna, Austria. ISBN 3–900,051–07-0, URL http://www.R-project.org/. Reif J C, Melchinger A E, Frisch M. Genetical and mathematical properties of similarity and dissimilarity coefficients applied in plant breeding and seed bank management[J]. Crop Science, 2005, 45(1): 1–7. Wright, S. 1978. Evolution and genetics of populations. Vol. IV. The Univ. of Chicago Press. (DOCX 62 kb) [file 12864_2017_3991_MOESM1_ESM.docx]

Fig. S1: PCoA analysis of the 95 samples reveals the outgroup *M. discoidea* to be clearly distinct from *M. recutita*

**

Colours: **green**: outgroup from *M. discoidea* (701_01, MD1_04, MD1_05, MD1_06); **red**: homogenous group of 14 tetraploid origins (007_02, 007_03, 007_04, 008_02, 008_04, 008_05, 011_01, 011_04, 011_05, 013_04, 013_06, 014_04, 014_05, 016_02, 016_04, 019_01, 019_03, 021_02, 021_03, 021_05, 022_01, 022_02, 022_04, 022_06, 023_01, 023_02, 023_03, 024_02, 024_03, 024_05, 024_07, 027_02, 027_03, 027_04, 032_01, 032_04, 032_05, 033_03, 033_05); **blue**: rather diverse group consisting of all remaining samples

Principal coordinate analysis (PCoA, Gower 1966) based on one Euclidean distance matrix termed modified Rogers’ distance (mRD, Wright 1978; Goodman and Stuber 1983; Reif et al. 2005) as another presentation of population structure. The specific algorithm was illustrated in Reif et al. 2005. Corresponding programming is executed in R (R Core Team 2013).

References:

Goodman M M, Stuber C W. Races of maize. 6: Isozyme variation among races of maize in Bolivia[R]. 1983.

Gower JC (1966) Some distance properties of latent root and vector methods used in multivariate analysis. Biometrika. 53(3-4):325-338.

R Core Team (2013). R: A language and environment for statistical computing. R Foundation for Statistical Computing, Vienna, Austria. ISBN 3-900051-07-0, URL http://www.R-project.org/.

Reif J C, Melchinger A E, Frisch M. Genetical and mathematical properties of similarity and dissimilarity coefficients applied in plant breeding and seed bank management[J]. Crop Science, 2005, 45(1): 1-7.

Wright, S. 1978. Evolution and genetics of populations. Vol. IV. The Univ. of Chicago Press.
