# Supplementary material for: Use of genotyping-by-sequencing to determine the genetic structure in the medicinal plant chamomile, and to identify flowering time and alpha-bisabolol associated SNP-loci by genome-wide association mapping
Source: BMC Genomics. 2017 Aug 10;18:599. doi: 10.1186/s12864-017-3991-0 (PMC5553732; doi:10.1186/s12864-017-3991-0)
Supplement: Supplementary file 2 — STRUCTURE* analysis (K = 4) including outgroup M. discoidea (last 4 samples, blue) reveals strict genetic separation from M. recutita. * The genotypes are represented by the vertical bars, whereas the different colours indicate the four genetic clusters. (DOCX 48 kb) [file 12864_2017_3991_MOESM2_ESM.docx]

Fig. S2: STRUCTURE* analysis (K=4) including outgroup *M. discoidea* (last 4 samples, blue) reveals strict genetic separation from *M. recutita*

* The genotypes are represented by the vertical bars, whereas the different colours indicate the four genetic clusters.
