# Supplementary material for: Use of genotyping-by-sequencing to determine the genetic structure in the medicinal plant chamomile, and to identify flowering time and alpha-bisabolol associated SNP-loci by genome-wide association mapping
Source: BMC Genomics. 2017 Aug 10;18:599. doi: 10.1186/s12864-017-3991-0 (PMC5553732; doi:10.1186/s12864-017-3991-0)
Supplement: Supplementary file 3 — Calculation of Delta K according to Evanno et al. (2005) by structure harvester for K = 1 to 15 (Earl and von Holdt 2012; K = number of clusters) determines values of 3 and 7 to fit the data best (DOCX 25 kb) [file 12864_2017_3991_MOESM3_ESM.docx]

Fig. S3: Calculation of Delta K according to Evanno et al. (2005) by structure harvester for K = 1 to 15 (Earl and von Holdt 2012; K=number of clusters) determines values of 3 and 7 to fit the data best
