# Supplementary material for: Use of genotyping-by-sequencing to determine the genetic structure in the medicinal plant chamomile, and to identify flowering time and alpha-bisabolol associated SNP-loci by genome-wide association mapping
Source: BMC Genomics. 2017 Aug 10;18:599. doi: 10.1186/s12864-017-3991-0 (PMC5553732; doi:10.1186/s12864-017-3991-0)
Supplement: Supplementary file 4 — STRUCTURE analysis assuming 3 clusters (K = 3). * The genotypes are represented by the vertical bars, whereas the different colours indicate the three genetic clusters (Additional file 5: Table S1). (DOCX 48 kb) [file 12864_2017_3991_MOESM4_ESM.docx]

Fig. S4: STRUCTURE analysis assuming 3 clusters (K=3)

* The genotypes are represented by the vertical bars, whereas the different colours indicate the three genetic clusters (Table S1).
