# Supplementary material for: Use of genotyping-by-sequencing to determine the genetic structure in the medicinal plant chamomile, and to identify flowering time and alpha-bisabolol associated SNP-loci by genome-wide association mapping
Source: BMC Genomics. 2017 Aug 10;18:599. doi: 10.1186/s12864-017-3991-0 (PMC5553732; doi:10.1186/s12864-017-3991-0)
Supplement: Supplementary file 5 — The different genetic clusters (K = 7) from the STRUCTURE analysis for the chamomile genotypes. (DOCX 18 kb) [file 12864_2017_3991_MOESM5_ESM.docx]

Table S1: The different genetic clusters (K=7) from the STRUCTURE analysis for the chamomile genotypes

| **genotype** | **cluster1** | **cluster2** | **cluster3** | **cluster4** | **cluster5** | **cluster6** | **cluster7** |
| --- | --- | --- | --- | --- | --- | --- | --- |
| 002_02 | 0.5426 | 0.3837 | 0.0138 | 0.0019 | 0.0011 | 0.0352 | 0.0217 |
| 002_04 | 0.5936 | 0.3392 | 0.0047 | 0.0021 | 0.0004 | 0.0591 | 0.0009 |
| 003_01 | 0.0492 | 0.2464 | 0.4509 | 0.1892 | 0.0618 | 0.001 | 0.0014 |
| 003_02 | 0.075 | 0.1943 | 0.2551 | 0.3342 | 0.1394 | 0.0007 | 0.0013 |
| 003_03 | 0.0194 | 0.2715 | 0.5838 | 0.0499 | 0.0318 | 0.0008 | 0.0428 |
| 003_04 | 0.0878 | 0.1925 | 0.4198 | 0.2557 | 0.038 | 0.0016 | 0.0046 |
| 004_01 | 0.2365 | 0.0002 | 0.211 | 0.0507 | 0.5008 | 0.0003 | 0.0005 |
| 004_03 | 0.1728 | 0.0269 | 0.3994 | 0.0391 | 0.3604 | 0.0005 | 0.0008 |
| 004_04 | 0.1614 | 0.003 | 0.3014 | 0.1737 | 0.3596 | 0.0006 | 0.0003 |
| 005_01 | 0.0011 | 0.3958 | 0.0109 | 0.004 | 0.0006 | 0.001 | 0.5866 |
| 005_02 | 0.0013 | 0.0161 | 0.1637 | 0.0013 | 0.0006 | 0.0006 | 0.8164 |
| 005_03 | 0.0016 | 0.0156 | 0.0886 | 0.0008 | 0.0002 | 0.0007 | 0.8925 |
| 005_04 | 0.002 | 0.0121 | 0.1746 | 0.0018 | 0.0006 | 0.0007 | 0.8082 |
| 006_01 | 0.0014 | 0.0969 | 0.0057 | 0.0013 | 0.0008 | 0.0006 | 0.8933 |
| 006_03 | 0.0092 | 0.294 | 0.0555 | 0.0032 | 0.0078 | 0.0013 | 0.629 |
| 006_04 | 0.0004 | 0.4376 | 0.0415 | 0.0002 | 0.0002 | 0.0003 | 0.5197 |
| 007_01 | 0.0014 | 0.4297 | 0.0041 | 0.0013 | 0.0005 | 0.001 | 0.562 |
| 007_02 | 0.0008 | 0.9674 | 0.0307 | 0.0002 | 0.0003 | 0.0004 | 0.0002 |
| 007_03 | 0.0002 | 0.9981 | 0.0007 | 0.0001 | 0.0002 | 0.0003 | 0.0004 |
| 008_02 | 0.0006 | 0.9375 | 0.0597 | 0.0004 | 0.0002 | 0.0006 | 0.001 |
| 008_04 | 0.0002 | 0.9989 | 0.0002 | 0.0001 | 0.0001 | 0.0003 | 0.0002 |
| 008_05 | 0.0022 | 0.9936 | 0.0008 | 0.0013 | 0.0006 | 0.0013 | 0.0002 |
| 009_02 | 0.0194 | 0.3789 | 0.3982 | 0.1321 | 0.0101 | 0.006 | 0.0552 |
| 009_03 | 0.0364 | 0.4751 | 0.2727 | 0.1044 | 0.1077 | 0.001 | 0.0026 |
| 010_02 | 0.0045 | 0.0007 | 0.1483 | 0.5992 | 0.2449 | 0.0015 | 0.0009 |
| 010_06 | 0.0013 | 0.0001 | 0.0003 | 0.4648 | 0.5317 | 0.0016 | 0.0002 |
| 011_01 | 0.0005 | 0.9976 | 0.0008 | 0.0002 | 0.0002 | 0.0004 | 0.0003 |
| 011_04 | 0.0002 | 0.9981 | 0.001 | 0.0001 | 0.0001 | 0.0003 | 0.0002 |
| 011_05 | 0.0005 | 0.9968 | 0.0014 | 0.0002 | 0.0005 | 0.0003 | 0.0003 |
| 013_06 | 0.0012 | 0.993 | 0.001 | 0.0023 | 0.001 | 0.0009 | 0.0006 |
| 014_04 | 0.0002 | 0.9988 | 0.0004 | 0.0001 | 0.0001 | 0.0003 | 0.0001 |
| 014_05 | 0.0005 | 0.997 | 0.0009 | 0.0003 | 0.0002 | 0.0007 | 0.0004 |
| 016_02 | 0.0729 | 0.8603 | 0.0018 | 0.0366 | 0.0256 | 0.0019 | 0.0008 |
| 016_04 | 0.0013 | 0.9922 | 0.0003 | 0.0026 | 0.0005 | 0.0017 | 0.0014 |
| 019_01 | 0.0002 | 0.999 | 0.0002 | 0.0001 | 0.0001 | 0.0003 | 0.0001 |
| 019_03 | 0.0005 | 0.9929 | 0.0018 | 0.0003 | 0.0003 | 0.0003 | 0.0039 |
| 021_02 | 0.0002 | 0.9984 | 0.0005 | 0.0002 | 0.0001 | 0.0004 | 0.0002 |
| 021_03 | 0.0002 | 0.9988 | 0.0003 | 0.0001 | 0.0001 | 0.0004 | 0.0001 |
| 021_05 | 0.0002 | 0.9969 | 0.0018 | 0.0001 | 0.0002 | 0.0003 | 0.0005 |
| 022_01 | 0.0006 | 0.9983 | 0.0001 | 0.0002 | 0.0003 | 0.0003 | 0.0002 |
| 022_02 | 0.0002 | 0.9969 | 0.0016 | 0.0003 | 0.0003 | 0.0005 | 0.0002 |
| 022_04 | 0.0011 | 0.995 | 0.0003 | 0.0004 | 0.0004 | 0.0021 | 0.0007 |
| 022_06 | 0.0003 | 0.9901 | 0.0086 | 0.0002 | 0.0003 | 0.0003 | 0.0002 |
| 023_01 | 0.0003 | 0.9984 | 0.0006 | 0.0001 | 0.0001 | 0.0003 | 0.0002 |
| 023_02 | 0.0002 | 0.9982 | 0.0008 | 0.0002 | 0.0001 | 0.0003 | 0.0002 |
| 023_03 | 0.0012 | 0.9883 | 0.0062 | 0.0009 | 0.0006 | 0.0007 | 0.0021 |
| 024_02 | 0.0002 | 0.999 | 0.0002 | 0.0001 | 0.0001 | 0.0003 | 0.0001 |
| 024_03 | 0.0008 | 0.9892 | 0.0088 | 0.0003 | 0.0004 | 0.0003 | 0.0002 |
| 024_05 | 0.0002 | 0.9974 | 0.0011 | 0.0004 | 0.0002 | 0.0004 | 0.0003 |
| 024_07 | 0.0002 | 0.9983 | 0.0008 | 0.0001 | 0.0002 | 0.0003 | 0.0001 |
| 026_01 | 0.0591 | 0.1881 | 0.3888 | 0.2033 | 0.1586 | 0.001 | 0.0011 |
| 026_04 | 0.0739 | 0.0841 | 0.2495 | 0.3663 | 0.2248 | 0.0006 | 0.0009 |
| 026_05 | 0.0716 | 0.1423 | 0.3463 | 0.2594 | 0.1781 | 0.0011 | 0.0013 |
| 027_02 | 0.0168 | 0.9615 | 0.0013 | 0.0094 | 0.008 | 0.0014 | 0.0016 |
| 027_04 | 0.0005 | 0.9977 | 0.0009 | 0.0002 | 0.0003 | 0.0003 | 0.0001 |
| 029_03 | 0.0218 | 0.0067 | 0.0012 | 0.9046 | 0.0008 | 0.0647 | 0.0002 |
| 029_04 | 0.0089 | 0.0013 | 0.0002 | 0.9575 | 0.0004 | 0.0316 | 0.0001 |
| 029_07 | 0.028 | 0.0085 | 0.0002 | 0.9132 | 0.0004 | 0.0489 | 0.0008 |
| 032_01 | 0.0004 | 0.997 | 0.0016 | 0.0002 | 0.0002 | 0.0003 | 0.0003 |
| 032_04 | 0.0008 | 0.9956 | 0.0007 | 0.0013 | 0.0004 | 0.0007 | 0.0005 |
| 032_05 | 0.0002 | 0.9988 | 0.0002 | 0.0003 | 0.0001 | 0.0003 | 0.0001 |
| 033_03 | 0.0005 | 0.9972 | 0.0006 | 0.0008 | 0.0005 | 0.0003 | 0.0001 |
| 033_05 | 0.0004 | 0.9984 | 0.0004 | 0.0002 | 0.0002 | 0.0003 | 0.0001 |
| 064_01 | 0.0004 | 0.0013 | 0.9974 | 0.0002 | 0.0001 | 0.0005 | 0.0001 |
| 064_03 | 0.0008 | 0.0018 | 0.9955 | 0.0002 | 0.0002 | 0.0006 | 0.0009 |
| 066_04 | 0.1395 | 0.2287 | 0.0607 | 0.3629 | 0.1672 | 0.0347 | 0.0064 |
| 066_07 | 0.1282 | 0.2001 | 0.1306 | 0.3556 | 0.1497 | 0.0327 | 0.0032 |
| 515_01 | 0.204 | 0.1418 | 0.2349 | 0.1913 | 0.2249 | 0.0014 | 0.0018 |
| 516_07 | 0.0005 | 0.0002 | 0.0001 | 0.0017 | 0.0045 | 0.9929 | 0.0001 |
| 715_04 | 0.0028 | 0.0014 | 0.3968 | 0.5654 | 0.0008 | 0.013 | 0.0198 |
| 717_03 | 0.6596 | 0.2724 | 0.0012 | 0.0012 | 0.0004 | 0.0631 | 0.0021 |
| 717_04 | 0.6918 | 0.219 | 0.0068 | 0.002 | 0.0006 | 0.0583 | 0.0214 |
| 721_03 | 0.0005 | 0.0011 | 0.9955 | 0.0005 | 0.0012 | 0.0003 | 0.0009 |
| 721_04 | 0.0025 | 0.0042 | 0.9468 | 0.0074 | 0.0012 | 0.001 | 0.0369 |
| 722_03 | 0.001 | 0.0175 | 0.9774 | 0.0013 | 0.0006 | 0.0007 | 0.0015 |
| 722_04 | 0.0002 | 0.0023 | 0.9953 | 0.0004 | 0.0003 | 0.0003 | 0.0012 |
