# Supplementary material for: Use of genotyping-by-sequencing to determine the genetic structure in the medicinal plant chamomile, and to identify flowering time and alpha-bisabolol associated SNP-loci by genome-wide association mapping
Source: BMC Genomics. 2017 Aug 10;18:599. doi: 10.1186/s12864-017-3991-0 (PMC5553732; doi:10.1186/s12864-017-3991-0)
Supplement: Supplementary file 6 — STRUCTURE* analysis with allele frequencies assumed to be independent among populations and for 7 clusters (K) leads to low population structure differentiation. * The genotypes are represented by the vertical bars, whereas the different colours indicate the seven genetic clusters. (DOCX 45 kb) [file 12864_2017_3991_MOESM6_ESM.docx]

Fig. S5: STRUCTURE* analysis with allele frequencies assumed to be independent among populations and for 7 clusters (K) leads to low population structure differentiation

* The genotypes are represented by the vertical bars, whereas the different colours indicate the seven genetic clusters.
