# Supplementary material for: Use of genotyping-by-sequencing to determine the genetic structure in the medicinal plant chamomile, and to identify flowering time and alpha-bisabolol associated SNP-loci by genome-wide association mapping
Source: BMC Genomics. 2017 Aug 10;18:599. doi: 10.1186/s12864-017-3991-0 (PMC5553732; doi:10.1186/s12864-017-3991-0)

Fig. S6: Average ploidy level for the groups of plants organized according to their number of main STRUCTURE-clusters (>= 9.5 %)


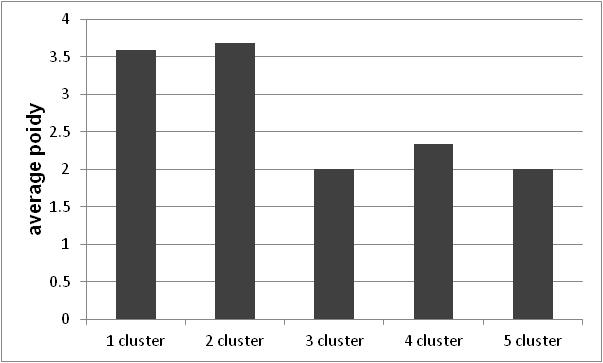

Supplement: Supplementary file 7 — Average ploidy level for the groups of plants organized according to their number of main STRUCTURE-clusters (> = 9.5%). (DOCX 35 kb) [file 12864_2017_3991_MOESM7_ESM.docx]
