# Supplementary material for: Use of genotyping-by-sequencing to determine the genetic structure in the medicinal plant chamomile, and to identify flowering time and alpha-bisabolol associated SNP-loci by genome-wide association mapping
Source: BMC Genomics. 2017 Aug 10;18:599. doi: 10.1186/s12864-017-3991-0 (PMC5553732; doi:10.1186/s12864-017-3991-0)
Supplement: Supplementary file 8 — STRUCTURE* analysis assuming 7 clusters (K = 7), organized according to Table 2. * The genotypes are represented by the vertical bars and the different origins are separated by vertical black lines, whereas the different colours indicate the seven genetic clusters (Additional file 5: Table S1). The top row indicates the geographical origin (upper) and ploidy (lower) for each sample according to Table 1 (DOCX 516 kb) [file 12864_2017_3991_MOESM8_ESM.docx]

Fig. S7: STRUCTURE* analysis assuming 7 clusters (K=7), organized according to table 2.


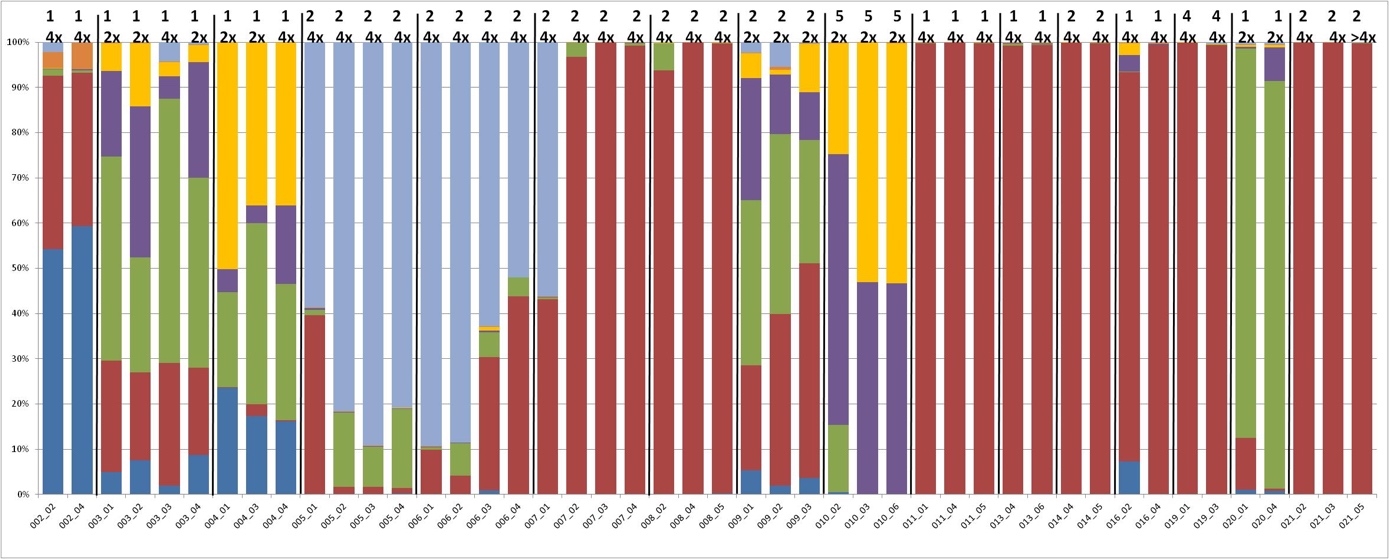


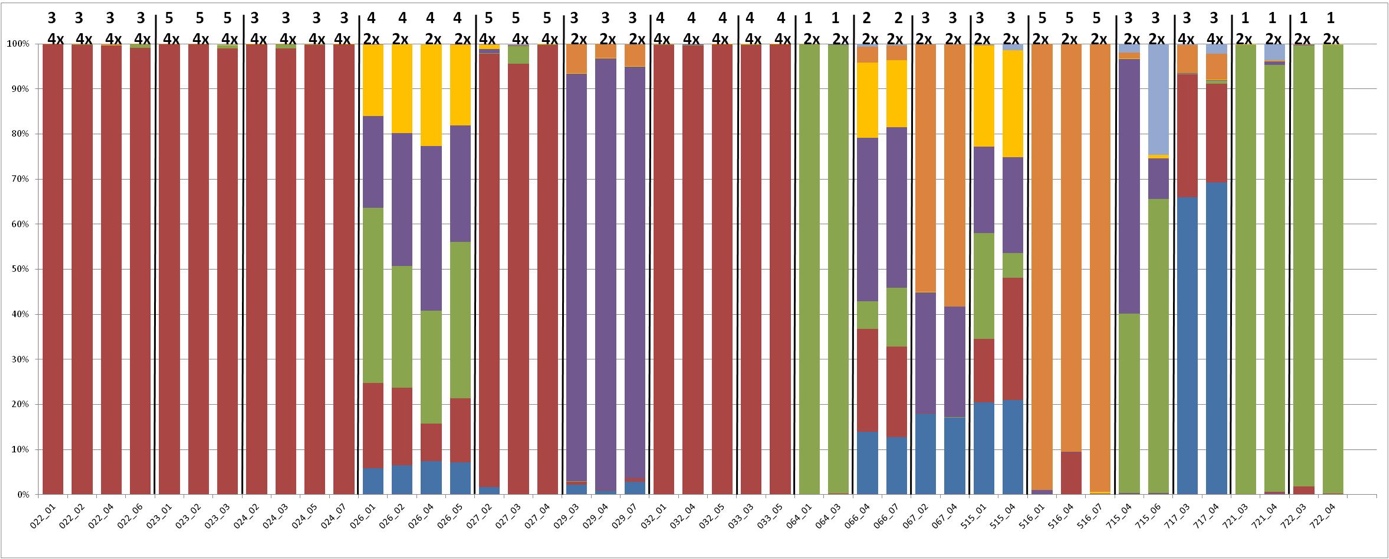


* The genotypes are represented by the vertical bars and the different origins are separated by vertical black lines, whereas the different colours indicate the seven genetic clusters (Table S1). The top row indicates the geographical origin (upper) and ploidy (lower) for each sample according to Table 1.
