# Supplementary material for: Use of genotyping-by-sequencing to determine the genetic structure in the medicinal plant chamomile, and to identify flowering time and alpha-bisabolol associated SNP-loci by genome-wide association mapping
Source: BMC Genomics. 2017 Aug 10;18:599. doi: 10.1186/s12864-017-3991-0 (PMC5553732; doi:10.1186/s12864-017-3991-0)
Supplement: Supplementary file 9 — PCoA analysis of the 91 samples M. recutita coloured according to (a) ploidy, (b) geographic origin, (c) flowering time. (a) ploidy. (b) geographic origin. (c) flowering time. PCoA performed as described in Additional file 1: Fig. S1. (a): red: diploid, green: tetraploid (b) code for geographic origin see Table 1 (c) start flowering from days after sowing (DAS): 1 (78 DAS - 107 DAS), 2 (108 DAS - 200 DAS), 3 (201 DAS - 260 DAS), 4 (261 DAS - 298 DAS), 1–2: short day conditions; 3–4: long day conditions. (DOCX 431 kb) [file 12864_2017_3991_MOESM9_ESM.docx]

Fig. S8: PCoA analysis of the 91 samples *M. recutita* coloured according to (a) ploidy, (b) geographic origin, (c) flowering time


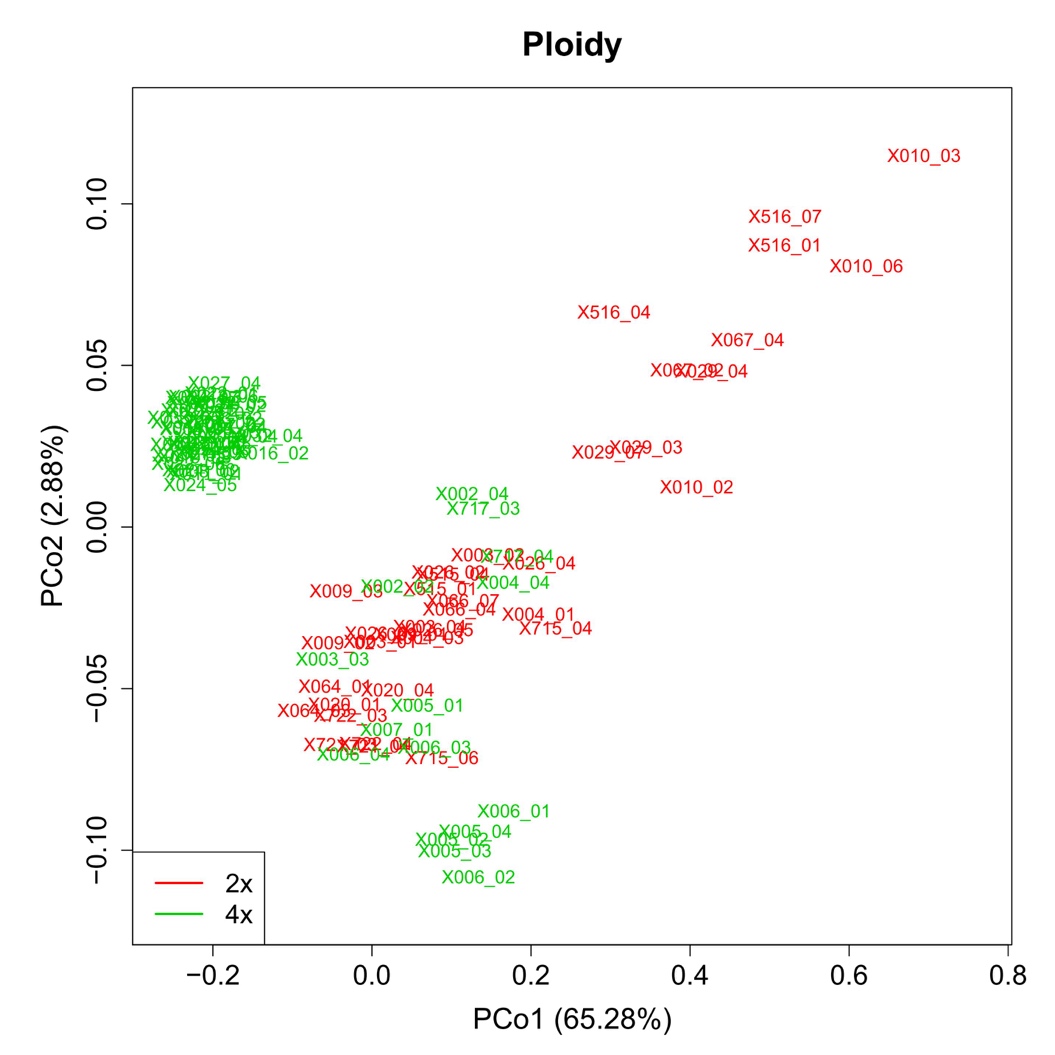
(a) ploidy

(b) geographic origin


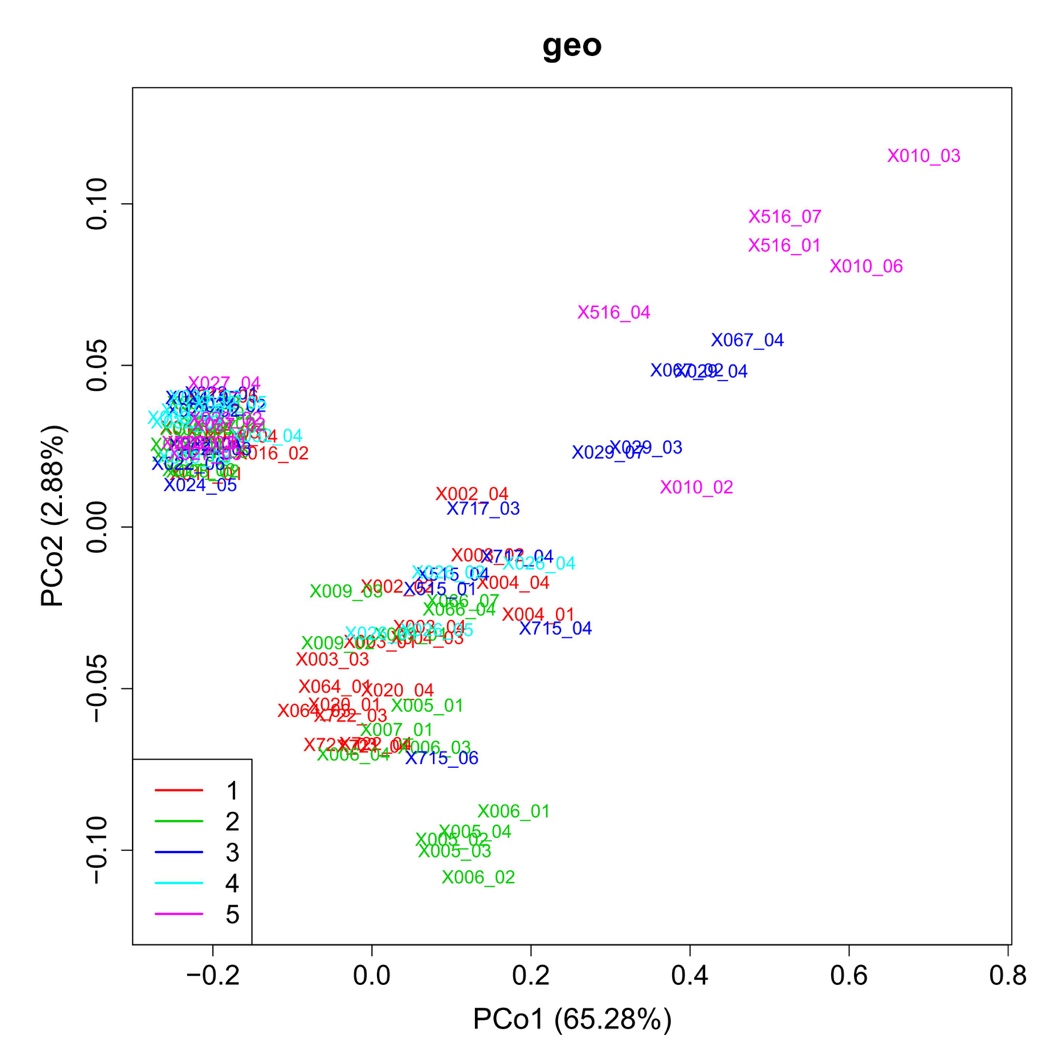


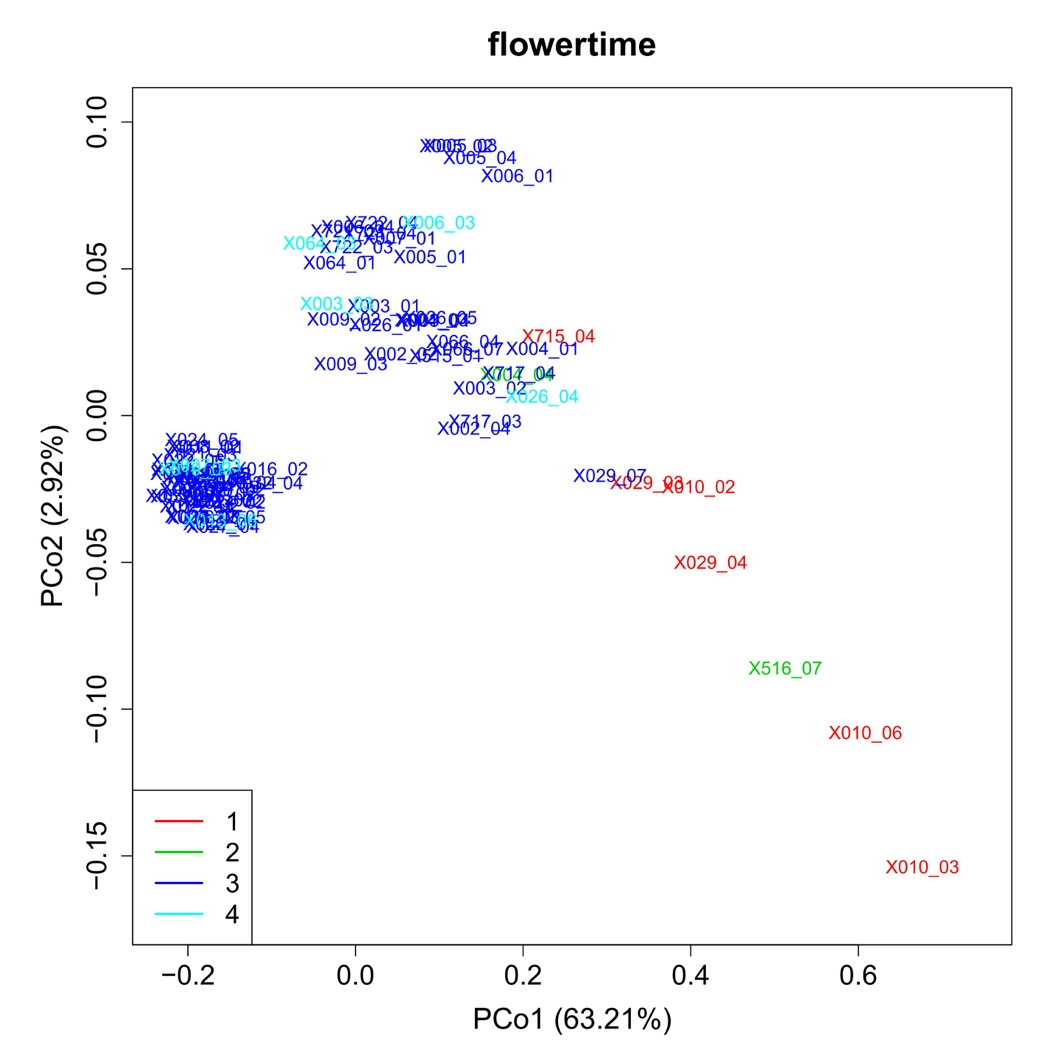


(c) flowering time

PCoA performed as described in Fig. S1

(a): red: diploid, green: tetraploid (b) code for geographic origin see Table 1 (c) start flowering from days after sowing (DAS): 1 (78 DAS - 107 DAS), 2 (108 DAS - 200 DAS), 3 (201 DAS - 260 DAS), 4 (261 DAS - 298 DAS), 1-2: short day conditions; 3-4: long day conditions
