# Supplementary material for: Use of genotyping-by-sequencing to determine the genetic structure in the medicinal plant chamomile, and to identify flowering time and alpha-bisabolol associated SNP-loci by genome-wide association mapping
Source: BMC Genomics. 2017 Aug 10;18:599. doi: 10.1186/s12864-017-3991-0 (PMC5553732; doi:10.1186/s12864-017-3991-0)
Supplement: Supplementary file 10 — Flowering time for diploid and tetraploid chamomile (DOCX 11 kb) [file 12864_2017_3991_MOESM10_ESM.docx]

Table S2: Flowering time for diploid and tetraploid chamomile

| Ploidy | **2x** | **4x** |
| --- | --- | --- |
| number samples | 27 | 51 |
| mean | 201.00 | 244.14 |
| standard deviation | 64.82 | 21.34 |
| Minimum value | 78.00 | 119.00 |
| Quartil1 | 182 | 239 |
| Median | 236.00 | 244.00 |
| Quartil3 | 239 | 252 |
| Maximum value | 260.00 | 285.00 |
