# Supplementary material for: Use of genotyping-by-sequencing to determine the genetic structure in the medicinal plant chamomile, and to identify flowering time and alpha-bisabolol associated SNP-loci by genome-wide association mapping
Source: BMC Genomics. 2017 Aug 10;18:599. doi: 10.1186/s12864-017-3991-0 (PMC5553732; doi:10.1186/s12864-017-3991-0)
Supplement: Supplementary file 11 — Genomic heritability: result from analysis 20 times with 5-fold cross validation (DOCX 13 kb) [file 12864_2017_3991_MOESM11_ESM.docx]

Table S3: Genomic heritability: result from analysis 20 times with 5-fold cross validation

| Trait | flowering time | bisabolol oxide_B | bisabolon oxide_A | alpha-bisabolol | bisabolol oxide_A |
| --- | --- | --- | --- | --- | --- |
| CV01 | 0.543322148 | 0.00254494 | 0.00031692 | 0.4637659 | 0.139357126 |
| CV02 | 0.445748623 | 0.023239201 | 0.003848141 | 0.4150842 | 0.102631081 |
| CV03 | 0.505868082 | 0.005059847 | 0.000731362 | 0.3786181 | 0.154570472 |
| CV04 | 0.477674507 | 0.001423732 | 0.008281163 | 0.5355068 | 0.11894853 |
| CV05 | 0.466670025 | 0.007483827 | 0.000801162 | 0.3765172 | 0.109961417 |
| CV06 | 0.45446591 | 0.001546449 | 0.00220598 | 0.3981033 | 0.139878415 |
| CV07 | 0.506921039 | 0.009006195 | 0.00848565 | 0.3588981 | 0.080821067 |
| CV08 | 0.492080359 | 0.007145805 | 5.39E-06 | 0.4804606 | 0.118858926 |
| CV09 | 0.551776896 | 0.000235767 | 0.009268472 | 0.3633469 | 0.068455907 |
| CV10 | 0.481740376 | 6.39E-07 | 0.015828455 | 0.3348409 | 0.130288302 |
| CV11 | 0.485552038 | 0.000749798 | 0.000160463 | 0.4449784 | 0.085500331 |
| CV12 | 0.403697536 | 0.010214053 | 0.005810437 | 0.3510463 | 0.118053481 |
| CV13 | 0.520639569 | 7.52E-05 | 0.009653481 | 0.4650174 | 0.074622059 |
| CV14 | 0.41478998 | 0.002700585 | 0.003408531 | 0.2882168 | 0.149208263 |
| CV15 | 0.400857283 | 0.041432753 | 0.007714956 | 0.4749193 | 0.125446537 |
| CV16 | 0.428583484 | 0.001207154 | 0.007820321 | 0.4220455 | 0.157900616 |
| CV17 | 0.446941411 | 9.30E-05 | 0.014753748 | 0.3286066 | 0.176992603 |
| CV18 | 0.505696523 | 4.97E-05 | 4.60E-06 | 0.2965693 | 0.099137665 |
| CV19 | 0.480256584 | 0.000525712 | 0.004554821 | 0.4613708 | 0.107952666 |
| CV20 | 0.513815154 | 0.000405186 | 6.07E-06 | 0.4542252 | 0.191554858 |
| mean | **0.476354876** | 0.005756977 | 0.005183006 | **0.40460688** | 0.122507016 |
| standard  deviation | 0.043533253 | 0.010085709 | 0.004911862 | 0.067566246 | 0.033295174 |
