# Supplementary material for: Use of genotyping-by-sequencing to determine the genetic structure in the medicinal plant chamomile, and to identify flowering time and alpha-bisabolol associated SNP-loci by genome-wide association mapping
Source: BMC Genomics. 2017 Aug 10;18:599. doi: 10.1186/s12864-017-3991-0 (PMC5553732; doi:10.1186/s12864-017-3991-0)
Supplement: Supplementary file 12 — Significant SNPs for the traits flowering time (ft, 9 SNPs) and alpha-bisabolol content (bis, 72 SNPs)*. * The SNPs for the traits flowering time (ft, 9 SNPs) and alpha-bisabolol content (bis, 72 SNPs) are contained in 5 and 60 different DNA-sequences, respectively. The sequences in the table are organized according to the p-value. ** Red letters in the sequence indicate the SNPs identified for this sequence, whereas bold red letters indicate the actual SNP for which the data are given in the corresponding line and for which the other alleles are listed in the fourth column. The single-letter IUPAC nucleotide code is used. (DOCX 23 kb) [file 12864_2017_3991_MOESM12_ESM.docx]

Table S4: Significant SNPs for the traits flowering time (ft, 9 SNPs) and alpha-bisabolol content (bis, 72 SNPs)*

| SNP No | DNA-sequence (seq.)** with SNPs (red), of which for one (**bold**) the alleles are listed right | seq. No | alleles | P–value | R^2^ |
| --- | --- | --- | --- | --- | --- |
| ft1 | AAGAAGGCAAC**A**ATATTGCAAATGGCAACAATGTATACAATATTAGGGGGTACCCTCGTAAACATTGGTGTCACATTTAGCACACAAG | 4585 | R,G | 5.87E-13 | 0.674 |
| ft2 | AAGAAGGCAACA**A**TATTGCAAATGGCAACAATGTATACAATATTAGGGGGTACCCTCGTAAACATTGGTGTCACATTTAGCACACAAG | 4585 | W,T | 5.87E-13 | 0.674 |
| ft3 | CTTCAATGTCAAAGTCCTTGTAGTATGCGTAAAAGGGTGCTTTGTTCCAATC**T**ATTTTTTCAAGCCCACCCCTTGTTGCCCAATCATCRGCTTCCCATAATGTTGAGTATACCCCCATT | 2445 | W | 2.88E-08 | 0.381 |
| ft4 | CTTCAATGTCAAAGTCCTTGTAGTATGCGTAAAAGGGTGCTTTGTTCCAATCTATTTTTTCAAG**C**CCACCCCTTGTTGCCCAATCATCRGCTTCCCATAATGTTGAGTATACCCCCATT | 2445 | Y | 2.88E-08 | 0.381 |
| ft5 | CGATCTCAAATTACTCGAGAAAAGCCAGATAATAATC**A**GTACTCCGGATAGATGGGATGCATTATCTCGCCGTT | 441 | Y | 4.89E-08 | 0.474 |
| ft6 | AAGAAGGCAACAATATTGCAAATGGCAACAATGTATACAATATTAGGGGGTACCCT**C**GTAAACATTGGTGTCACATTTAGCACACAAG | 4585 | Y | 1.22E-07 | 0.423 |
| ft7 | TTTTTGAAGCGCAGAAGCACTCGAAGATCTAAATAGCGTACG**G**ATATCGGTCCCAAACGTCAAAGCATCTT | 3029 | R | 1.74E-07 | 0.605 |
| ft8 | TTTTTGAAGCGCAGAAGCACTCGAAGATCTAAATAGCGTACGGATATC**G**GTCCCAAACGTCAAAGCATCTT | 3029 | S,K,C,Y | 1.74E-07 | 0.605 |
| ft9 | TTTTCTTTTAGCTTTGGATAGCACAAGATCCTTGGAAACGCAAACAC**C**AAGTGCCACCCACTTGGAATCAATACAAAGTGAACATGCATTGCATATTTTACCGTGGCTTGT | 6153 | S | 5.06E-07 | 0.358 |
| bis1 | TTTTACATATGCAACAATCAGGACTTAAGATAG**C**ACAGTGGAAGAAATAGTTATGTGGCTCTTTCCATAGACAAC-GCGGAACAAATT | 4316 | Y, | 2.28E-15 | 0.674 |
| bis2 | CAGTAGACTGTTCCTAGTTTAAATGAAATTAAAATT**T**TCAGTTAGGTCTCATAACCAAAAACCATCCGATTATAACGAAACTAGTCTATGCGGATGGAGAAGGAATATCAACAACATTT | 1278 | W | 2.30E-14 | 0.523 |
| bis3 | ATAGCTTCAATAGTTCAGCTGACTGTTGTTTTAGCAATGGATCACAACCACTCTGGAT**A**ACAAGGAGCAGTTTTGCTGAGATCGGA | 3994 | M | 3.32E-14 | 0.616 |
| bis4 | TGATATCTTTCTATGGAAGGACAAGAAGGTTACTGCTGGTGTGCTTGGTTTTGCCACTCTGATTTGGGT**G**CTTTTTGAATTGGTTGAGTATCACTTGCTTACACTAGTATGCCACACTCTRAT | 6654 | S, R, A | 4.77E-14 | 0.649 |
| bis5 | CAGAATCAAGATCATCAGCATAAGAACTACAGCAAGAAAACTGACGACGATAAAACAAACTATT**C**TTCATTGTCAGATCCGCCAAAATT | 4792 | Y | 1.42E-11 | 0.428 |
| bis6 | AC**G**TATCCGTTTGAAGTTGTTAGACGACAGCTTCAGTTGCAAGTTCGTGCCACGAAAATGAGTGCAG | 3420 | R | 2.71E-11 | 0.451 |
| bis7 | TAGAGGTAAACTAATCTTCAAAAAT**C**CTCATGAGCTTTGGAAGATTCTCCCATTGATTCATCAGGTTTAGTTTTCACTCTTTGGTAAAACAAAAGATAT | 1810 | Y | 4.46E-10 | 0.410 |
| bis8 | TTATTATAAGGAC**G**GGCATTCGGACATTTGTCCATCTGATCCAAGAATGCCTCCGCCAAATCCAACTCCAGGCCATTACTGCAACCTGCTGATAT | 3495 | S | 4.46E-10 | 0.410 |
| bis9 | ATTCTTCCTCTTTACATCATGGAGCTCCTGAAATTCAATATGTAAGCATAACGTAACY**A**CACAACCTAGAATTACATTTAATAAGGA | 4038 | R | 4.46E-10 | 0.410 |
| bis10 | AGGAAAGGACTTCACATTGGAAACTGGGAAAACTACC**G**CTCAATATGCAGGTAAGGCGGCAGGTGCGTTGAAAGATACAGCTCTCAGC | 4558 | R | 4.46E-10 | 0.410 |
| bis11 | CATTTCAATAAGAACAAGCTTTCAGGCGGAATTCCTGAACA**A**CTTTTCAGTGACAACATGGTGCTGATACATGTGTAAGCTGAAAACCGAACCCT | 614 | R | 4.46E-10 | 0.410 |
| bis12 | AAGAATGGAAAGAAGGCAGACAGTTCTTCTGA**A**GAAAGTTCCGAAGATGAGAGTGATTCTGAGGAAGAGGTAAAAGCWG | 912 | R | 4.46E-10 | 0.410 |
| bis13 | TCCAGATGCAAAGTTTAGTCC**C**CCGAGTATWGCTCGCCCACGAGTTCTTGCATAAGGTGGAATGTTGTTTCGAAATCCTAGRAGTT | 2208 | Y | 4.46E-10 | 0.410 |
| bis14 | TCCAGATGCAAAGTTTAGTCC**C**CCGAGTATWGCTCGCCCACGAGTTCTTGCATAAGGTGGAATGTTGTTTCGAAATCCTAGRAGTT | 2208 | A | 4.46E-10 | 0.410 |
| bis15 | AATAGCACTAAAGGATGAAGCTTCTAAAGCTCAGGATAATGTCGATTCAACTTTGCTTGCTATTGAAAAGATC**G**AGAATGAAGAAATT | 3073 | R | 2.09E-09 | 0.431 |
| bis16 | ATCATAATAACTGGTTCCCGCACTGATCCCAACTGGAGGTTCTTTAACTCGGATCCAATATTTGCATACCTTAATTCTGATTTAGCT**G**GTGGGGGY | 3081 | R | 2.09E-09 | 0.431 |
| bis17 | AGTATTCACTGGATACATAGCAGAAGGTTCATGCTTATTTTCTCCTTTATAATCATGCACAGACC**T**AGCTTCTCTATCATATTTAACACCTTGATACAAGTCTCGTGATTCT | 3092 | K | 2.09E-09 | 0.431 |
| bis18 | AAACTCCAACAGATAGTGGGAACTTTACAACCACTTCAATCCCCTTTAGCTGTGCTCGTGATTCTCCATTAGAGTGCAACTCTGTACCAAACACTAATT**T**G | 1851 | Y | 8.84E-09 | 0.473 |
| bis19 | ATATTTGTCAACCAAGGGTCGGAATACAGGGTCTTCAAGAAGAGCCTTGTC**A**GTAGGCAGAGATCGGAAGAGCGGTTCAGCAGGAA | 722 | R,G | 1.03E-08 | 0.403 |
| bis20 | TGCAAAAGAGGCCCGCAAGTCWAACTTATGTCAAA**G**TTATAGTGATTTCGGTTCTCGTCAAGATTTAAGTGTGARAAAAYCAGAAGATGGTATACTTGTGGAGGAGTT | 256 | A,R | 1.60E-08 | 0.429 |
| bis21 | TGCAAAAGAGGCCCGCAAGTCWAACTTATGTCAAAGTTATAG**T**GATTTCGGTTCTCGTCAAGATTTAAGTGTGARAAAAYCAGAAGATGGTATACTTGTGGAGGAGTT | 256 | Y | 1.60E-08 | 0.429 |
| bis22 | TGCAAAAGAGGCCCGCAAGTCWAACTTATGTCAAAGTTATAGTGATTTCGGTTCTCGTCAAGAT**T**TAAGTGTGARAAAAYCAGAAGATGGTATACTTGTGGAGGAGTT | 256 | G,K | 1.60E-08 | 0.429 |
| bis23 | TCCAGCGTCATAGACCAATCCGGGGTCGACAGCCTTTAAGGGGTTGATATGTCCAGAACCATA**T**GCAAATTCAGCATCTGAGTTTTTTATTGGACTCATTGGT | 5460 | C,Y | 1.61E-08 | 0.398 |
| bis24 | TCGACCAGATGGGTCAAG**A**TTTTTGGACCAAGATGCGTCCCACTCCACTGCTTTGGCGGTACTGCATGCCACACTTGGACCGCCTGGAACGGTTARCCTT | 4194 | R,G | 4.15E-08 | 0.368 |
| bis25 | TAGACTGATGGGTTGCAAACTGAAGGACCTATCAGTCGCTCTATGNACCAACAAAAATCAGTCTGCTTCCGATGACACGGTCCAA**A**TTTCAAC | 1379 | T | 5.32E-08 | 0.317 |
| bis26 | TAGACTGATGGGTTGCAAACTGAAGGACCTATCAGTCGCTCTATGNACCAACAAAAATCAGTCTGCTTCCGATGACACGGTCCAAATT**T**CAAC | 1379 | C | 5.32E-08 | 0.317 |
| bis27 | AACAGTAGTGCGTGGTTAGGCACAGCGGTTCTTGTTACAAACTTAAGAAACTTCCCATTAAGTCGTGGTACAGTTGCTGGC**C**TTTTAAAAGGCTACATTGCCCTTAGT | 1998 | T | 5.32E-08 | 0.317 |
| bis28 | AACAGTAGTGCGTGGTTAGGCACAGCGGTTCTTGTTACAAACTTAAGAAACTTCCCATTAAGTCGTGGTACAGTTGCTGGCCTTT**T**AAAAGGCTACATTGCCCTTAGT | 1998 | A | 5.32E-08 | 0.317 |
| bis29 | TCAATGACACCGCGTTAGAAGATGCAATCAAAACCATAGAAATGGATGGATATAAAGTTATAGG**G**ACACATGACAAAAATACAGAAGAAGAGCCAATTTCACTGGAAACT | 5681 | A | 5.32E-08 | 0.317 |
| bis30 | TCAATGACACCGCGTTAGAAGATGCAATCAAAACCATAGAAATGGATGGATATAAAGTTATAGGG**A**CACATGACAAAAATACAGAAGAAGAGCCAATTTCACTGGAAACT | 5681 | T | 5.32E-08 | 0.317 |
| bis31 | ATCCATGTCTCCAAGGAGTTTTTATTCATTTCCTAATTCGGGTTTGAGAAGTTTTCCAATGGG**G**TTAAGCAGCAG | 1698 | T,R, A | 5.32E-08 | 0.317 |
| bis32 | CGCCCAAGCAATCCGTCC**G**GTTGTTTTTAAAAAGTCACTAGAAAATATTAAACTAACATCTCNACAGGCTTANGAATGGGTCATGCAAACTGAA | 6449 | C,K | 5.32E-08 | 0.317 |
| bis33 | ACAGTTTATGGAAAGCGACACAGTTGCAATCGT**A**GGCCCACAATCSTCAGTCGTAGCTCACATTATATCTCACGTAGCWAACGAACTCCAAGTCCCGCTCTTATCATTC | 1139 | W | 5.32E-08 | 0.317 |
| bis34 | AGTATCGAAAATGCTAACATTGGGCTCGAGCAAATTAACCATCTTGCTTCTGCAGA**T**GGTGACACAATGCAAAGAATT | 1418 | Y | 5.32E-08 | 0.317 |
| bis35 | AACGAAGTACATAWACGCCAACGTAAACATTAACATGCATTCCAATACCTTTTTCTATTGTGTCAATTACC**C**ATAGATAACATATACAAACTTTGCCAGTGTGGGGTATCT | 2223 | M | 5.32E-08 | 0.317 |
| bis36 | AATCATTGGATCAGCTCGTTGAAATTAAAGCACGAGCTTCCATGTTCGAAAAGA**T**CTTGTCGAGATTTGCWG | 2380 | Y | 5.32E-08 | 0.317 |
| bis37 | AATCATTGGATCAGCTCGTTGAAATTAAAGCACGAGCTTCCATGTTCGAAAAGATCTTGTCGAGATT**T**GCWG | 2380 | Y | 5.32E-08 | 0.317 |
| bis38 | AGGCCTTCACGATGGTA**G**TCGCTAATTGTGTTGTCAACAAGTATGACTAAGTCTCGTGTTGACCCTGACAT---ACGGATGCTTTGG | 2487 | R | 5.32E-08 | 0.317 |
| bis39 | TTTGTCTTGATATATTCCTGAACAGGAAAACAAAACAAA**C**TAGTCAGGTTACAGAATAYCACAACCCTCAT | 2489 | Y | 5.32E-08 | 0.317 |
| bis40 | TTTGTCTTGATATATTCCTGAACAGGAAAACAAAACAAACTAG**T**CAGGTTACAGAATAYCACAACCCTCAT | 2489 | S | 5.32E-08 | 0.317 |
| bis41 | GATGCAGGAAAAGA**A**GTGGTGGTTGCTGATGCCATCAAGGCCCAATCAGCAGAGATCGGAAGAGCGGTTCAGCAGGAATGCCGAGACCG | 2735 | R | 5.32E-08 | 0.317 |
| bis42 | TTGAGCTTCAGGTTCAGGTTGT**G**GTTGAGATGTTGTTTGTTCAGCTTTATAAGAATTAGGAATGTACTTCCAACAACGCTTGTGAACACCGCTAATTTTCTTTGGAGCAT | 2892 | R | 5.32E-08 | 0.317 |
| bis43 | AAGTCATTAAGTGAAGCAAGG**A**GATGACTGCTCTATAGATTAATAAAGCCTGCAAACTTGGTTGATCTTTTT | 2962 | R | 5.32E-08 | 0.317 |
| bis44 | AAAGACCCAAAAGAAGGATACAGGAC**A**ATAGTCGAGAACCAACCTGTTTACATACACCCCGGTAGCGCGCTTTTCAATAGAAACCCTGATTGGGTCATCTACCATGA | 3249 | M | 5.32E-08 | 0.317 |
| bis45 | ACAATCTTTCA**G**TTGCAAGCACCCAATCTACATGATGTGAGAMACATATATTATACAGCTAGTTCAT | 3345 | R | 5.32E-08 | 0.317 |
| bis46 | ACCTGGAGTTACTGTGCCCACTGATCATAGCAAGATAACTAATCAAATGGCAA**C**ACCAGCTT | 4510 | M | 5.32E-08 | 0.317 |
| bis47 | AATGGCTAACTTTC**T**CGAGTATGATATCTACGACCTGGAGTTAACGTCGGTTCAAGAYAACACTGATTTAAGGAAGCWG | 4535 | M | 5.32E-08 | 0.317 |
| bis48 | AATTCGG**A**ATAGCCACCAATCACCGCCACCCAATTTTCCTTCAATAAGATATTGCWG | 6 | R | 5.32E-08 | 0.317 |
| bis49 | CTAATGTTTATCATCAA**T**GGGTAGGCCGATGCATGTTTCAAGTGTCAGGTGAATGCCTTTCATGTAATACGAAAGTTGTTTTACGCAAGT | 5258 | Y | 5.32E-08 | 0.317 |
| bis50 | AAAACCTAACCCGGCTAGTGTTATCCCAA-CACCCTTAATCCTATATAACGAGTCCTTAATTAAAAACTAGAACAT-AAAAA**T**GCCATAGTTGAGCATACTAAACCTTTTAAATTGACTTGGAGT | 5419 | K | 5.32E-08 | 0.317 |
| bis51 | AATGTTTCGCCAGATGAACAAGATTATTTGTTAC**A**TTTACTGCTTGAGTCAGAAAACGAAGCWG | 5696 | R | 5.32E-08 | 0.317 |
| bis52 | CTTGATAAACTTAGAATCCGTGTAATGTGATTGTTAAAAAAT**G**AATTCAGTAGGC-TTTTTG-ATTGATGTAATATCTAGTAATGAGGTTTTAACAATTATTTGGCTGATTTGTGGTTTAAACTTGMTATAT | 5824 | S | 5.32E-08 | 0.317 |
| bis53 | ACA**G**AAATGGTAAGTGGAGATGGTCACACACTCTGATTTAGCTTATTCAGTCTGGGAGACAATTTATAT | 6080 | R | 5.32E-08 | 0.317 |
| bis54 | AGTACTTGGGACCTAACCCAGGACTTCCTTCTATATCG**G**ACCAACCAACCCAGGACTCAAGCTCATTGGACTCTTCAGCTGGTCCTGTT | 6366 | K | 5.32E-08 | 0.317 |
| bis55 | ATATGTGATCAGGGCGTGAATTTAGCACTGGGAGAGTATTAGGTGTGTCAGCTTTACTAGGATCTTTCTTCCTTCGGCCACGCTTTGCACATTGT**A**CGGATGGTAAGAAGCTCATTT | 6401 | R | 5.32E-08 | 0.317 |
| bis56 | CWGCCTCGCTTTCTCATTCCATGAAGACTGATGTTAATCTT**A**ATTGCAGAACAGTCTTATGACCGAATTCCAAATTTT | 6440 | M | 5.32E-08 | 0.317 |
| bis57 | CWGCCTCGCTTTCTCATTCCATGAAGACTGATGTTAATCTTAATTGCAGAACAGTCTTATGAC**C**GAATTCCAAATTTT | 6440 | M | 5.32E-08 | 0.317 |
| bis58 | CWGCCTCGCTTTCTCATTCCATGAAGACTGATGTTAATCTTAATTGCAGAACAGTCTTATGACCGAATTCC**A**AATTTT | 6440 | R | 5.32E-08 | 0.317 |
| bis59 | AGGAGTGGCNACGGGTGGAGCTAGAACAAGAGTGGCTACGGGTGGAGCTACAACTGGAGTGGCTACGGGTGGAGCTACAACAG**G**AGGGGC | 6670 | R | 5.32E-08 | 0.317 |
| bis60 | CGGGAGAGGG**T**TTTCGGGGGCTAAACGGTAATCTATCGACATGATAACACAACCTACTTTACTAGCTAAACGTGCAAGAAACTCATGGTAACAACTCCAT | 1732 | K | 1.06E-07 | 0.377 |
| bis61 | TGAAGAGGCMTTTGAACRTGGCTCCGACACAATGGGCAAGTG**G**AATGGTCTTTTAACCAGAACTCGATGCACCCAAGGTGGAATTGGACTCCATGATT | 5751 | R | 1.06E-07 | 0.377 |
| bis62 | TAGTGTTATTAGCTTCTCCACATGTTCGAATACAA**C**ACATAGTCGTTTAAGGTCTTCGATTATCTCTATATCCCCAGGTAAMTTGTTT | 5946 | Y | 1.10E-07 | 0.404 |
| bis63 | TGACTTTGGAATTG**C**GTTTATGAAGGCAACTTTCTTCACCTTCTTGAATGGAGCCACCTGAGAGATGCAAGTAATTACAGTCACACGATTTGTGTT | 477 | Y, T | 1.29E-07 | 0.385 |
| bis64 | ATGCTTATTTTTGACCACACGAGATGCAGTTTCAAACTC**A**GATGCAACCATTGTAACCTTTCCCATTGCATATGCATTTTTTATCGAGTCYTGAAGTTTTTGGCCTCCTGAT | 6386 | R | 1.65E-07 | 0.527 |
| bis65 | TCGATTGCAAGATCTTTTGGG**G**CGAATGACTGTCGAAGAAAAAATTGGTCAAATGGTTCAAATTGAAAGGATTGCAGAGATCGGAAGAG | 6623 | R | 2.72E-07 | 0.253 |
| bis66 | TCGATTGCAAGATCTTTTGGGGCGAATGAC**T**GTCGAAGAAAAAATTGGTCAAATGGTTCAAATTGAAAGGATTGCAGAGATCGGAAGAG | 6623 | K | 2.72E-07 | 0.253 |
| bis67 | TGCAGTTTTGGTGCATCTTTGTTCTGGCGATCTGAAGTATCTGGT**T**GAGGCTCAAGAACTTGGAGTGATGGGTATACTTGTGGATTT | 3223 | Y | 2.98E-07 | 0.313 |
| bis68 | CGCGTATATAGCTAAACAGTTGCTCACCATTTGTGACAAGTCTTTTAGCTTCACTTCCATTTGTTTCTTCAC**A**TACCCATTTTCCACCTCAGCTAAACCATCAGTACACGTGTCCTGGTTAGT | 3619 | R | 3.00E-07 | 0.338 |
| bis69 | TTTTGAGAGGCGTTTCCAACAGGTTTTGGTGGCTGAGCCGAGTGTTCCTGATAC**C**ATAAGTATTCTTAGAGGGTTGAAAGAAAGATATGAAGG | 5721 | Y | 3.00E-07 | 0.338 |
| bis70 | TGGTGGTATCGGCCAACCATTATCTCTTTTG**G**TAAAGATGTCCCCTCTGGTTTCGGACCTTCACCTTTACGATATAGCCAATGTCAAGGGAGT | 6598 | R | 3.00E-07 | 0.338 |
| bis71 | AGGAGTGGCNACGGGTGGAGCTAGAACAAGAGTGGCTACGGGTGGAGCTACAACTGGAGTGGCTACGGGTGGAGCTACAACAGG**A**GGGGC | 6670 | R | 3.28E-07 | 0.255 |
| bis72 | ATGCAAACTAGACCAGTGATCATACCATTAACTGCACCAAT**G**ACTGAAGGTTTTTTGAANACAATTATGTCTAAGATGAGCCACGTGAGGAGGCTTGTA | 3218 | A, R | 5.18E-07 | 0.314 |

* The SNPs for the traits flowering time (ft, 9 SNPs) and alpha-bisabolol content (bis, 72 SNPs) are contained in 5 and 60 different DNA-sequences, respectively. The sequences in the table are organized according to the p-value.

** Red letters in the sequence indicate the SNPs identified for this sequence, whereas bold red letters indicate the actual SNP for which the data are given in the corresponding line and for which the other alleles are listed in the fourth column. The single-letter IUPAC nucleotide code is used.
