# Supplementary material for: Use of genotyping-by-sequencing to determine the genetic structure in the medicinal plant chamomile, and to identify flowering time and alpha-bisabolol associated SNP-loci by genome-wide association mapping
Source: BMC Genomics. 2017 Aug 10;18:599. doi: 10.1186/s12864-017-3991-0 (PMC5553732; doi:10.1186/s12864-017-3991-0)
Supplement: Supplementary file 13 — BLAST alignment results* for 5 and 13 sequences harbouring SNPs significantly associated with flowering time (FT) and alpha-bisabolol, respectively. * BLAST alignment results threshold: BLAST-score of > = 80 with a sequence identity of > = 80%/75% and an E-value <1.33 × 10^−13^; (in brackets): results below the threshold (minimum BLAST-score of >55 with a sequence identity of > = 75% and an E-value <1 × 10^−4^. The blastn algorithm was used for the analysis of the data, since the results obtained from tblastx were similar to the ones from blastn. Tblastx achieved not more hits above the significance threshold than blastn at the NCBI database. ** A more general annotation approach, an HMM (hidden Markov model) search through the Protein families database, did also not yield any results for sequence 6153. *** tblastx. The BLAST alignment of the 5 sequences harbouring significantly associated SNPs to FT (Additional file 12: Table S4) against the NCBI nucleotide collection of flowering plants identified 2 sequences with significant hits to potential candidate genes in multiple plant species (BLAST-score threshold see above, with a sequence identity of > = 80%). Below the significance threshold, for 3 sequences an alignment to plant species could be done, but not for sequence 6153. (1) Jankowsky E. RNA Helicases at work: binding and rearranging. Trends in biochemical sciences. 2011;36(1):19–29. doi:10.1016/j.tibs.2010.07.008. (2) Kurasawa K, Matsui A, Yokoyama R, Kuriyama T, Yoshizumi T, Matsui M, Suwabe K, Watanabe M, Nishitani K. The AtXTH28 gene, a xyloglucan endotransglucosylase/hydrolase, is involved in automatic self-pollination in Arabidopsis thaliana. Plant Cell Physiol. 2009; 50(2):413–22. doi: 10.1093/pcp/pcp003. From the above 13 alpha-bisabolol associated sequences, one has been described as microsatellite sequence (1379). For the remaining 12 sequences, specific gene products were described in multiple plant species: “722” ascorbate peroxidase 2-like protein, 3 [file 12864_2017_3991_MOESM13_ESM.docx]

Table S5: BLAST alignment results* for 5 and 13 sequences harbouring SNPs significantly associated with flowering time (FT) and alpha-bisabolol, respectively

| sequence No. | Described as* | in No of plant species | Suggested functional role |
| --- | --- | --- | --- |
| 441 | (DExH-box ATP-dependent RNA / and U5 small nuclear ribonucleoprotein 200 kDa helicase mRNA) | (5) | binding and remodeling RNA or RNA-protein complexes^1^ |
| 2445 | xyloglucan endotransglucosylase/hydrolase | 30 | involved in self-pollination in *Arabidopsis thaliana^2^* |
| 3029 | (phytochrome C) | (4) |  |
| 4585 | unkown protein (protein kinase / salt-induced ABC1 kinase) | 9 (2) |  |
| 6153 | -** | -** |  |
| 1732 | (predicted: probable carboxylesterase 6) | (5) | - |
| 1998 | predicted: nuclear fusion defective 4-like (LOC109841189) protein | 2 | female gametophyte development^3^ |
| 6386 | uncharacterized protein (lipase)*** | 18 (1)*** | - |
| 1379 | microsatellite sequence | 2 | - |
| 722 | ascorbate peroxidase 2-like protein | 3 | directly involved in the protection of plant cells against adverse environmental conditions^4^ |
| 1139 | glutamate receptor | 2 | response to pathogens/ pests attack^5^ |
| 3218 | ammonium transporter 3 member 1-like | 2 |  |
| 3223 | U-box domain-containing protein 13 | 3 |  |
| 3223 | armadillo/beta-catenin repeat family protein | 3 | involved in stress responses^6^ |
| 3249 | probable pre-mRNA-splicing factor ATP-dependent RNA helicase | 23 |  |
| 4194 | mostly asparagine synthetase | 60 |  |
| 5721 | chaperone protein ClpB1/ heat shock protein | 42 | heat shock protein required for acclimation to high temperatures^7^ |
| 6598 | malate dehydrogenase, chloroplastic-like | 19 |  |
| 6654 | mainly reticulon-like protein B2 | 24 |  |

* BLAST alignment results threshold: BLAST-score of >=80 with a sequence identity of >=80%/75% and an E-value < 1.33 x 10^-13^; (in brackets): results below the threshold (minimum BLAST-score of >55 with a sequence identity of >=75% and an E-value < 1 x 10^-4^. The blastn algorithm was used for the analysis of the data, since the results obtained from tblastx were similar to the ones from blastn. Tblastx achieved not more hits above the significance threshold than blastn at the NCBI database.

** A more general annotation approach, an HMM (hidden Markov model) search through the Protein families database, did also not yield any results for sequence 6153.

*** tblastx

The BLAST alignment of the 5 sequences harbouring significantly associated SNPs to FT (Table S4) against the NCBI nucleotide collection of flowering plants identified 2 sequences with significant hits to potential candidate genes in multiple plant species (BLAST-score threshold see above, with a sequence identity of >=80%). Below the significance threshold, for 3 sequences an alignment to plant species could be done, but not for sequence 6153.

(1) Jankowsky E. RNA Helicases at work: binding and rearranging. *Trends in biochemical sciences*. 2011;36(1):19-29. doi:10.1016/j.tibs.2010.07.008.

(2) Kurasawa K, Matsui A, Yokoyama R, Kuriyama T, Yoshizumi T, Matsui M, Suwabe K, Watanabe M, Nishitani K. The AtXTH28 gene, a xyloglucan endotransglucosylase/hydrolase, is involved in automatic self-pollination in *Arabidopsis* *thaliana*. Plant Cell Physiol. 2009; **50(2)**:413-22. doi: 10.1093/pcp/pcp003.

From the above 13 alpha-bisabolol associated sequences, one has been described as microsatellite sequence (1379). For the remaining 12 sequences, specific gene products were described in multiple plant species: “722” ascorbate peroxidase 2-like protein, 3 species; “1139” glutamate receptor, 2 species; “1732” predicted: probable carboxylesterase 6, 5 species; “1998” predicted: nuclear fusion defective 4-like (NFD4; LOC109841189) protein, 2 species; “3218” ammonium transporter 3 member 1-like, 2 species; , “3223” U-box domain-containing protein 13 and armadillo/beta-catenin repeat family protein, 3 species; “3249” probable pre-mRNA-splicing factor ATP-dependent RNA helicase, 23 species; “4194” mostly asparagine synthetase, 60 species; “5721” chaperone protein ClpB1/ heat shock protein, 42 species; “6386” lipase, 1 species; “6598” malate dehydrogenase, chloroplastic-like, 19 species; “6654”, mainly reticulon-like protein B2, 24 species.

(3) Portereiko MF, Sandaklie-Nikolova L, Lloyd A, Dever CA, Otsuga D, Drews GN **NUCLEAR FUSION DEFECTIVE1 encodes the Arabidopsis RPL21M protein and is required for karyogamy during female gametophyte development and fertilization.** Plant Physiol. 2006; 141:957-965.

(4) Caverzan A, Passaia G, Rosa SB, Ribeiro CW, Lazzarotto F, Margis-Pinheiro M. Plant responses to stresses: Role of ascorbate peroxidase in the antioxidant protection. Genetics and Molecular Biology. 2012;35 (4 Suppl):1011-1019.

(5) Forde BG, Roberts MR. Glutamate receptor-like channels in plants: a role as amino acid sensors in plant defence? *F1000Prime Reports*. 2014;6:37. doi:10.12703/P6-37.

(6) Sharma M, Pandey A, Pandey GK. β-catenin in plants and animals: common players but different pathways. *Frontiers in Plant Science*. 2014;5:143. doi:10.3389/fpls.2014.00143.

(7) Lee U, Rioflorido I, Hong SW, Larkindale J, Waters ER, Vierling E.: 2007. The Arabidopsis ClpB/Hsp100 family of proteins: chaperones for stress and chloroplast development. Plant J. Jan;49(1):115-27.
