# Supplementary material for: Use of genotyping-by-sequencing to determine the genetic structure in the medicinal plant chamomile, and to identify flowering time and alpha-bisabolol associated SNP-loci by genome-wide association mapping
Source: BMC Genomics. 2017 Aug 10;18:599. doi: 10.1186/s12864-017-3991-0 (PMC5553732; doi:10.1186/s12864-017-3991-0)
Supplement: Supplementary file 14 — BLAST aligment (megablast) against the sunflower genome (https://www.heliagene.org/HanXRQ-SUNRISE/: Helianthus annuus XRQ genome assembly. The chamomile sequence names in bold letters were aligned by BLAST against NCBI nucleotide collection of flowering plants, and for them gene products were described in multiple plant species above the alignment threshold (Additional file 13: Table S5). (DOCX 23 kb) [file 12864_2017_3991_MOESM14_ESM.docx]

Table S6: BLAST aligment (megablast) against the sunflower genome (<https://www.heliagene.org/HanXRQ-SUNRISE/>: *Helianthus annuus* XRQ genome assembly

| **sunflower chromosome** | **Sequence**  **number chamomile** | **sequence start** | **sequence end** | **e-value** | **Sequence**  **identity [%]** |
| --- | --- | --- | --- | --- | --- |
| Chamomile sequences associated with flowering time | | | | | |
| HanXRQChr04 | 4585 | 174126635 | 174126550 | 6.00E-21 | 88.37 |
| HanXRQChr06 | **2445** | 34322776 | 34322699 | 3.00E-06 | 79.75 |
| HanXRQChr11 | **2445** | 13874489 | 13874371 | 4.00E-34 | 89.08 |
|  | 3029 | 35642178 | 35642108 | 2.00E-09 | 83.10 |
| HanXRQChr12 | **2445** | 99630995 | 99631111 | 1.00E-34 | 89.74 |
|  | 441 | 17053377 | 17053440 | 2.00E-05 | 81.25 |
| HanXRQChr14 | **2445** | 131996395 | 131996277 | 6.00E-38 | 90.76 |
| HanXRQChr15 | **2445** | 42287993 | 42288109 | 3.00E-36 | 90.60 |
| HanXRQChr17 | **2445** | 199842625  24210030 | 199842509  24210127 | 1.00E-29  6.00E-08 | 87.18  77.78 |
| Chamomile sequences associated with alpha-bisabolol | | | | | |
| HanXRQChr01 | 3994 | 8603678 | 8603747 | 1.00E-13 | 87.14 |
|  | 5751 | 76647930  76665168  76667128  79118562  79120490  79122990  79124953 | 76647850  76665098  76667048  79118482  79120417  79122920  79124873 | 3.00E-05  0.0003  6.00E-07  1.00E-08  2.00E-07  0.0003  6.00E-07 | 77.78  78.87  79.01  80.25  81.08  78.87  79.01 |
|  | 614 | 117787796 | 117787890 | 3.00E-19 | 85.26 |
| HanXRQChr02 | 3420 | 28253015  101752367 | 28253081  101752433 | 2.00E-10  2.00E-15 | 85.07  89.55 |
|  | **6598** | 90526199 | 90526110 | 4.00E-13 | 82.22 |
|  | **722** | 100742737 | 100742787 | 2.00E-11 | 92.16 |
|  | **3218** | 143982262 | 143982360 | 6.00E-12 | 79.80 |
| HanXRQChr03 | **3249** | 2013216 | 2013313 | 4.00E-14 | 81.63 |
|  | 4535 | 133292758  133370051 | 133292833  133370123 | 3.00E-09  5.00E-16 | 81.58  87.67 |
|  | 2892 | 138611094 | 138611203 | 9.00E-26 | 86.36 |
|  | 3073 | 167523935 | 167523848 | 2.00E-10 | 80.68 |
| HanXRQChr04 | 3619 | 26256514 | 26256630 | 1.00E-14 | 79.49 |
|  | 2892 | 86497719 | 86497610 | 9.00E-26 | 86.36 |
|  | **5721** | 170221668 | 170221613 | 1.00E-07 | 85.71 |
|  | **3218** | 177517671 | 177517619 | 8.00E-06 | 84.91 |
| HanXRQChr05 | **722** | 21040389 | 21040333 | 4.00E-13 | 91.23 |
|  | **3223** | 30638417 | 30638335 | 1.00E-12 | 83.13 |
|  | **4194** | 38579590 | 38579674 | 3.00E-10 | 81.18 |
|  | 1998 | 195938215  197524668 | 195938114  197524566 | 1.00E-14  1.00E-13 | 81.55  80.77 |
|  | 2487 | 210532656 | 210532738 | 3.00E-14 | 84.34 |
| HanXRQChr06 | 1810 | 6563191 | 6563269 | 8.00E-06 | 79.75 |
|  | **1139** | 103209915 | 103210022 | 3.00E-30 | 88.89 |
| HanXRQChr07 | 6623 | 37582974 | 37583045 | 2.00E-11 | 84.72 |
|  | 3994 | 65705547  65726007 | 65705501  65725961 | 6.00E-06  0.0003 | 87.23  85.11 |
|  | 5460 | 85849241  86899590 | 85849335  86899692 | 2.00E-12  1.00E-08 | 81.05  77.88 |
|  | 3619 | 100952762 | 100952878 | 3.00E-16 | 80.34 |
| HanXRQChr08 | 477 | 36097723 | 36097665 | 7.00E-06 | 83.05 |
|  | 6623 | 71704513 | 71704581 | 2.00E-06 | 81.43 |
|  | 3994 | 81716088 | 81716134 | 6.00E-06 | 87.23 |
|  | 5946 | 97575990 | 97575908 | 2.00E-16 | 85.54 |
|  | **4194** | 98663403  151634793 | 98663337  151634877 | 1.00E-08  3.00E-10 | 83.58  81.18 |
|  | 1998 | 127657934 | 127658041 | 1.00E-14 | 80.56 |
|  | 2892 | 151485780 | 151485889 | 4.00E-24 | 85.45 |
| HanXRQChr09 | 4535 | 112350815 | 112350889 | 9.00E-09 | 81.33 |
|  | 6401 | 129067946 | 129068051 | 3.00E-25 | 86.92 |
|  | 2208 | 177492610 | 177492525 | 5.00E-17 | 84.88 |
|  | **5721** | 188775575  195379293 | 188775483  195379250 | 2.00E-26  1.00E-07 | 90.32  90.91 |
|  | **6598** | 190642744 | 190642836 | 9.00E-10 | 79.57 |
|  | 1998 | 203363472 | 203363563 | 9.00E-06 | 77.17 |
| HanXRQChr10 | 6386 | 62794579 | 62794469 | 1.00E-13 | 79.28 |
|  | 6654 | 120681312  164794198 | 120681431  164794117 | 6.00E-28  4.00E-10 | 85.83  81.71 |
|  | 6623 | 162340560  240019620 | 162340628  240019552 | 2.00E-06  2.00E-06 | 81.43  81.43 |
|  | **1139** | 192823041 | 192822933 | 1.00E-09 | 77.68 |
|  | 2489 | 222217304 | 222217349 | 6.00E-05 | 86.96 |
|  | 5751 | 240785464 | 240785383 | 7.00E-11 | 81.71 |
|  | **3249** | 245127600 | 245127496 | 4.00E-19 | 83.96 |
| HanXRQChr11 | 3994 | 10792929 | 10792990 | 0.0003 | 80.65 |
|  | 256 | 25045422 | 25045337 | 1.00E-08 | 79.31 |
|  | 6623 | 25616199  146497718 | 25616256  146497764 | 2.00E-05  7.00E-06 | 82.76  87.23 |
|  | 477 | 77385088 | 77385148 | 6.00E-17 | 93.44 |
| HanXRQChr12 | 5751 | 36633248 | 36633167 | 3.00E-09 | 80.49 |
|  | 1851 | 67070559 | 67070659 | 2.00E-17 | 83.17 |
|  | **722** | 69465077 | 69465132 | 6.00E-11 | 89.47 |
|  | 6440 | 122174068 | 122174131 | 7.00E-10 | 85.94 |
| HanXRQChr13 | 6654 | 39206262 | 39206151 | 8.00E-12 | 78.57 |
|  | 1418 | 50902429  50916716 | 50902491  50916778 | 1.00E-06  1.00E-06 | 82.54  82.54 |
|  | 4535 | 104172475  104198002  104219432  104242296  104327871  104338986 | 104172550  104198077  104219507  104242371  104327946  104339061 | 0.0003  0.0003  5.00E-06  5.00E-06  3.00E-09  5.00E-06 | 77.63  77.63  78.95  78.95  81.58  78.95 |
|  | **4194** | 108562057 | 108561973 | 3.00E-10 | 81.18 |
|  | 6386 | 120176062 | 120176172 | 3.00E-05 | 74.77 |
|  | 6623 | 169556730 | 169556806 | 1.00E-07 | 80.52 |
|  | 3081 | 178007897 | 178007830 | 2.00E-07 | 82.61 |
|  | 3073 | 196461989 | 196462067 | 2.00E-06 | 79.75 |
| HanXRQChr14 | 3495 | 11067881 | 11067944 | 3.00E-10 | 85.94 |
|  | **4194** | 32028813 | 32028868 | 0.0004 | 82.14 |
|  | 3994 | 123218507 | 123218437 | 6.00E-11 | 84.72 |
|  | 6654 | 136573819 | 136573938 | 3.00E-31 | 87.50 |
|  | 5460 | 164350984  164356616 | 164350882  164356518 | 1.00E-23  2.00E-16 | 86.67  83.17 |
| HanXRQChr15 | 2892 | 5610887 | 5610958 | 2.00E-06 | 80.82 |
|  | **4194** | 20360048  64799945 | 20359949  64799879 | 2.00E-22  6.00E-12 | 86.00  86.57 |
|  | 5460 | 109704024 | 109703922 | 3.00E-15 | 81.55 |
|  | 3420 | 116425035 | 116424977 | 4.00E-06 | 83.05 |
|  | **3223** | 144143782 | 144143864 | 1.00E-07 | 79.52 |
| HanXRQChr16 | **5721** | 17345795  17432589 | 17345704  17432680 | 1.00E-32  3.00E-29 | 94.57  92.39 |
|  | 6386 | 60776996 | 60777079 | 1.00E-14 | 84.52 |
|  | **4194** | 121899005 | 121899071 | 3.00E-10 | 85.07 |
|  | 6366 | 154693497 | 154693575 | 2.00E-05 | 78.48 |
|  | 5460 | 171409503 | 171409554 | 3.00E-05 | 84.62 |
|  | 1732 | 172581528 | 172581429 | 1.00E-28 | 90.00 |
| HanXRQChr17 | 1732 | 201763397 | 201763496 | 6.00E-27 | 89.00 |

The chamomile sequence names in bold letters were aligned by BLAST against NCBI nucleotide collection of flowering plants, and for them gene products were described in multiple plant species above the alignment threshold (Table S5).
