# Supplementary material for: Use of genotyping-by-sequencing to determine the genetic structure in the medicinal plant chamomile, and to identify flowering time and alpha-bisabolol associated SNP-loci by genome-wide association mapping
Source: BMC Genomics. 2017 Aug 10;18:599. doi: 10.1186/s12864-017-3991-0 (PMC5553732; doi:10.1186/s12864-017-3991-0)
Supplement: Supplementary file 15 — Alpha-bisabolol in relation to the average heterozygosity for all polymorphic SNPs for the chamomile genotypes. The high alpha-bisabolol rich genotypes displayed an elevated level of heterozygosity, but more genotypes with the same level of heterozygosity contained no alpha-bisabolol (R2 = 0.056). The underlying data are listed in Additional file 16: Table S7. The two datasets used: (1) demultiplexed fasta-file of the barcoded reads for each genotype and. (2) the matrix for the filtered 6495 SNPs will be made publicly available after acceptance via e!DAL (http://edal.ipk-gatersleben.de) with a proprietary DOI, and during the revision process sent on request by the corresponding author. (DOCX 55 kb) [file 12864_2017_3991_MOESM15_ESM.docx]

Fig. S9: Alpha-bisabolol in relation to the average heterozygosity for all polymorphic SNPs for the chamomile genotypes


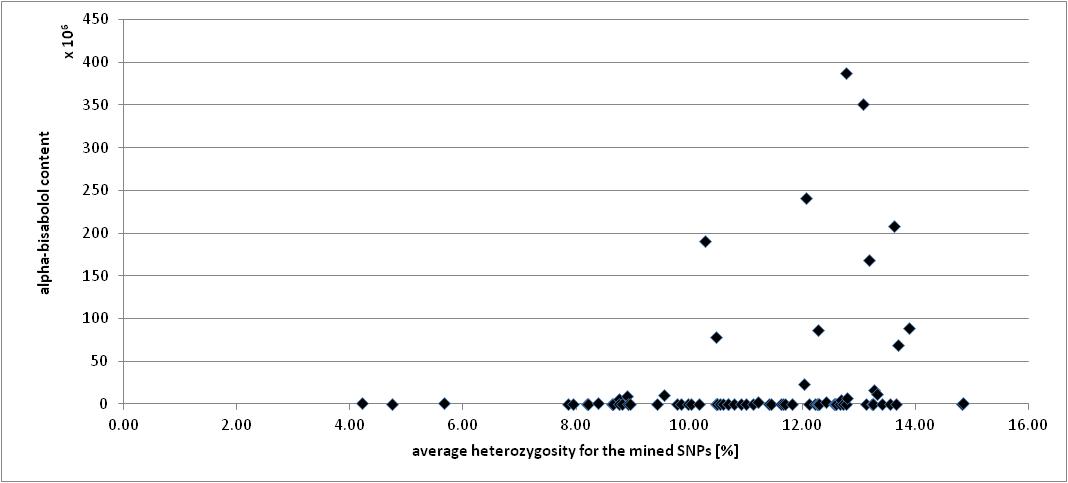


The high alpha-bisabolol rich genotypes displayed an elevated level of heterozygosity, but more genotypes with the same level of heterozygosity contained no alpha-bisabolol (R2=0.056). The underlying data are listed in Table S7.
