# Supplementary material for: Use of genotyping-by-sequencing to determine the genetic structure in the medicinal plant chamomile, and to identify flowering time and alpha-bisabolol associated SNP-loci by genome-wide association mapping
Source: BMC Genomics. 2017 Aug 10;18:599. doi: 10.1186/s12864-017-3991-0 (PMC5553732; doi:10.1186/s12864-017-3991-0)
Supplement: Supplementary file 16 — Number of SNPs showing heterozygosity and alpha-bisabolol content for the single chamomile genotypes. 1 In relation to 44,468 variable sites (polymorphic SNPs) mined by the pyRAD pipeline before filtering. 2 all values: × 106 (DOCX 16 kb) [file 12864_2017_3991_MOESM16_ESM.docx]

Table S7: Number of SNPs showing heterozygosity and alpha-bisabolol content for the single chamomile genotypes

| genotype | heterozygotic sites | % heterozygosity**^1^** | alpha-bisabolol  content**^2^** |
| --- | --- | --- | --- |
| 064_01 | 1877 | 4.22 | 1.41 |
| 004_01 | 2112 | 4.75 | 0.00 |
| 064_03 | 2522 | 5.67 | 1.13 |
| 721_03 | 3494 | 7.86 | 0.00 |
| 066_04 | 3536 | 7.95 | 0.00 |
| 722_03 | 3640 | 8.19 | 0.00 |
| 516_07 | 3652 | 8.21 | 0.00 |
| 721_04 | 3732 | 8.39 | 0.71 |
| 722_04 | 3838 | 8.63 | 0.00 |
| 009_03 | 3847 | 8.65 | 0.00 |
| 003_01 | 3895 | 8.76 | 5.33 |
| 515_01 | 3897 | 8.76 | 0.00 |
| 004_03 | 3920 | 8.82 | 0.00 |
| 715_04 | 3960 | 8.91 | 9.49 |
| 004_04 | 3962 | 8.91 | 0.00 |
| 029_04 | 3981 | 8.95 | 0.00 |
| 029_07 | 4197 | 9.44 | 0.00 |
| 029_03 | 4250 | 9.56 | 10.04 |
| 032_05 | 4351 | 9.78 | 0.00 |
| 026_01 | 4384 | 9.86 | 0.00 |
| 009_02 | 4437 | 9.98 | 0.00 |
| 066_07 | 4458 | 10.03 | 0.00 |
| 003_02 | 4520 | 10.16 | 0.00 |
| 002_02 | 4572 | 10.28 | 190.98 |
| 717_03 | 4653 | 10.46 | 78.79 |
| 016_04 | 4658 | 10.47 | 0.00 |
| 026_05 | 4663 | 10.49 | 0.00 |
| 024_05 | 4666 | 10.49 | 0.00 |
| 011_05 | 4691 | 10.55 | 0.00 |
| 003_04 | 4710 | 10.59 | 0.00 |
| 024_02 | 4753 | 10.69 | 0.00 |
| 010_02 | 4754 | 10.69 | 0.00 |
| 026_04 | 4800 | 10.79 | 0.00 |
| 016_02 | 4853 | 10.91 | 0.00 |
| 010_06 | 4895 | 11.01 | 0.00 |
| 003_03 | 4944 | 11.12 | 0.00 |
| 022_01 | 4983 | 11.21 | 1.87 |
| 021_03 | 5077 | 11.42 | 0.00 |
| 032_04 | 5089 | 11.44 | 0.00 |
| 011_01 | 5167 | 11.62 | 0.00 |
| 013_06 | 5182 | 11.65 | 0.00 |
| 023_03 | 5198 | 11.69 | 0.00 |
| 021_02 | 5253 | 11.81 | 0.00 |
| 019_03 | 5349 | 12.03 | 23.22 |
| 005_02 | 5362 | 12.06 | 241.47 |
| 008_04 | 5384 | 12.11 | 0.00 |
| 024_03 | 5435 | 12.22 | 0.00 |
| 008_02 | 5448 | 12.25 | 0.00 |
| 019_01 | 5455 | 12.27 | 0.00 |
| 005_01 | 5461 | 12.28 | 86.42 |
| 033_05 | 5470 | 12.30 | 0.00 |
| 033_03 | 5524 | 12.42 | 1.68 |
| 024_07 | 5582 | 12.55 | 0.21 |
| 022_06 | 5589 | 12.57 | 0.00 |
| 014_04 | 5597 | 12.59 | 0.00 |
| 022_04 | 5634 | 12.67 | 0.16 |
| 005_04 | 5637 | 12.68 | 4.45 |
| 008_05 | 5657 | 12.72 | 0.00 |
| 032_01 | 5677 | 12.77 | 0.00 |
| 005_03 | 5681 | 12.78 | 387.08 |
| 014_05 | 5688 | 12.79 | 7.14 |
| 002_04 | 5812 | 13.07 | 351.12 |
| 023_02 | 5836 | 13.12 | 0.00 |
| 006_01 | 5862 | 13.18 | 168.52 |
| 027_02 | 5886 | 13.24 | 0.00 |
| 021_05 | 5889 | 13.24 | 0.00 |
| 022_02 | 5898 | 13.26 | 16.76 |
| 007_02 | 5924 | 13.32 | 11.76 |
| 027_04 | 5958 | 13.40 | 0.00 |
| 011_04 | 6021 | 13.54 | 0.00 |
| 717_04 | 6059 | 13.63 | 208.66 |
| 023_01 | 6068 | 13.65 | 0.00 |
| 007_01 | 6088 | 13.69 | 68.74 |
| 006_03 | 6173 | 13.88 | 89.10 |
| 006_04 | 6586 | 14.81 | 0.00 |
| 007_03 | 6599 | 14.84 | 1.19 |

**^1^** In relation to 44468 variable sites (polymorphic SNPs) mined by the pyRAD pipeline before filtering.

**^2^** peak area; all values: x 10^6^
